# The Impact of *Citrus*-Tea Cofermentation Process on Chemical Composition and Contents of Pu-Erh Tea: An Integrated Metabolomics Study

**DOI:** 10.3389/fnut.2021.737539

**Published:** 2021-09-17

**Authors:** Ya Xu, Pu-Lin Liang, Xue-Lian Chen, Ming-Jiong Gong, Liang Zhang, Xiao-Hui Qiu, Jing Zhang, Zhi-Hai Huang, Wen Xu

**Affiliations:** ^1^Key Laboratory of Quality Evaluation of Chinese Medicine of the Guangdong Provincial Medical Products Administration, The Second Clinical College of Guangzhou University of Chinese Medicine, Guangzhou, China; ^2^State Key Laboratory of Tea Plant Biology and Utilization, Anhui Agricultural University, Hefei, China; ^3^Guangdong Provincial Key Laboratory of Clinical Research on Traditional Chinese Medicine Syndrome, Guangdong Provincial Hospital of Chinese Medicine, Guangzhou, China; ^4^Guangzhou Key Laboratory of Chirality Research on Active Components of Traditional Medicine, Guangzhou, China

**Keywords:** Ganpu tea, co-fermentation, UHPLC-QE-Orbitrap MS, polyphenols, metabolomics, pu-erh tea

## Abstract

Ganpu tea, an emerging pu-erh compound tea, which is cofermented with the peel of *Citrus reticulata* “Chachi,” has been widely favored by Chinese consumers due to its potential health effects and distinct flavor and taste. So far, the influence of this cofermentation procedure on the chemical profile of pu-erh tea has barely been addressed yet. In this work, an ultra-high-performance liquid chromatography-Q Exactive Orbitrap mass spectrometry (UHPLC-QE Orbitrap MS)-based qualitative and quantitative method combined with multivariate analysis was conducted to comprehensively investigate the chemical changes in pu-erh tea after cofermented with *Citrus* peel. A total of 171 compounds were identified based on a three-level strategy, among which seven phenolic acids, 11 flavan-3-ols, and 27 flavonoids and flavonoid glycosides were identified from pu-erh tea for the first time. Eighty-nine main constituents were selected for further quantitative analysis using a validated method. Both the principal component analysis (PCA) of untargeted metabolomics and orthogonal partial least squares discriminant analysis (OPLS-DA) models of targeted components revealed the significant chemical profile disparity between the raw pu-erh tea and Ganpu tea. It showed that *Citrus* tea cofermentation process significantly decreased the total contents of phenolic acids, flavan-3-ols, and flavonoid aglycones, while most of the quercetin glycosides and myricetin glycosides as well as the vitexin were significantly increased. In addition, hesperidin, a flavonoid glycoside only existed in *Citrus*, was first found in pu-erh tea after cofermented with *Citrus*. This study clearly profiled the chemical composition and content changes of pu-erh tea after cofermented with *Citrus* peel, which revealed that *Citrus* tea cofermentation process further accelerated the fermentation of pu-erh tea and improved the unique flavor of tea.

## Introduction

Pu-erh tea (pu'er, or pu-er, PE), is a representative postfermented tea (dark tea) in China. Marked by its distinct flavor and morphological appearance, PE has become increasingly popular and received more and more attention for its health benefits (1, 2). The unique flavor of PE is attributed to the large-leaf tea species of *Camellia sinensis* var. *assamica* that is traditionally planted in Pu-er and Xishuangbanna of Yunnan province, as well as to the way in which the tea is handled. Generally, the raw Pu-erh tea (*Sheng* pu-erh) is made from the minimally processed large-leaf variety, and compressed into shapes. The *Sheng* pu-erh would be then subjected to aging naturally for several years, resulting in a slow fermentation. However, this time-consuming method was substituted in industry scale by a style of processing approach called pile-fermentation, which involves the application of heat and moisture, as well as the inoculation of the tea leaves with beneficial bacteria to expedite the natural aging process. This pile-fermented dark tea is called *Shou* pu-erh tea (ripen pu-erh tea).

**Figure 1:**
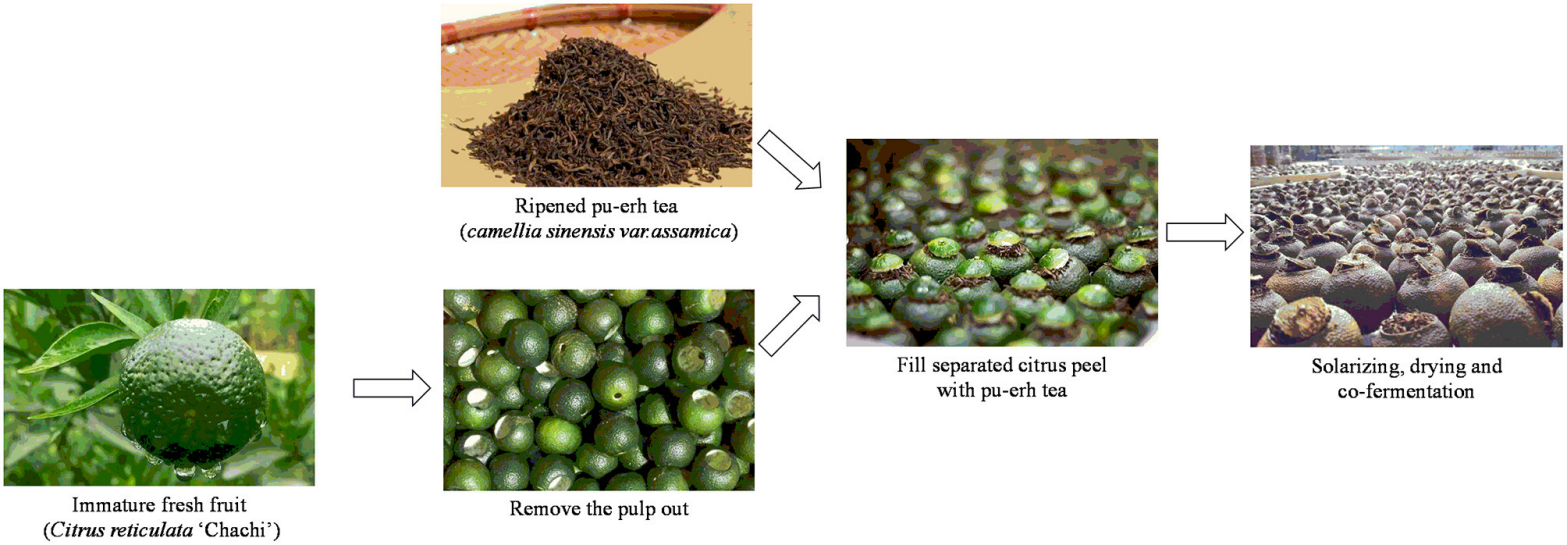
The manufacturing process of Ganpu tea.

In recent years, a new kind of pu-erh tea namely Ganpu tea has been widely popular among the Chinese market. It is a compound tea that is cofermented with the *Citrus* peel from a specific variety of *Citrus reticulata* “Chachi.” The preparation process of Ganpu tea can be briefly described as follows: First, the meat of fresh *Citrus* fruit (*C. reticulata* “Chachi”) was removed, and the formed peel cavity was filled with PE, and then the encapsulated PE in the *Citrus* peel was solarized, dried, aged, and/or pile-fermented together (Figure 1). As the tea aging and fermenting occurs within a closed environment formed by *Citrus* peel, the major ingredients of *Citrus* and tea interact and hence form the special flavor of Ganpu tea (3, 4). Ganpu tea has a long history since Tang dynasty when tea processed with *Citrus* peel was first recorded, and now the market share of Ganpu tea in China has increased significantly. Currently, a variety of Ganpu teas that are made of differential *Citrus* peels at different maturity (immature, near mature, mature) and various pu-erh tea materials are available in the Chinese tea market.

This new form of dark tea attracted scientific attention in terms of its unique flavor, chemical property, and functions (4, 5). There are some studies on the analysis of aroma components of Ganpu tea (1), but the chemical profile of non-volatile components during the cofermentation processing is seldom addressed. In recent years, high-throughput analytical strategies that coupled liquid chromatography combined with mass spectrometry (LC-MS) (6) and proton nuclear magnetic resonance (1H-NMR) (7) with metabolomics and other multivariate statistical methods (8) are intensively applied for the studies concerning the chemical composition of *C. reticulata* “Chachi” and pu-erh tea. Sui Xiao et al. (9) identified and analyzed 104 water-soluble components of Ganpu tea and their variation trend during sun-drying processing based on ultra-high-performance liquid chromatography quadrupole time-of-flight mass spectrometry (UHPLC-Q-TOF/MS) technology. Q Exactive-Orbitrap-MS/MS (QE Orbitrap-MS/MS) combines high-performance quadrupole precursor selection with high-resolution, accurate mass Orbitrap detection to deliver high performance and tremendous versatility. One hundred and forty-five chemical components of pu-erh tea were characterized based on the ultra-high-performance liquid chromatography-Q Exactive Orbitrap mass spectrometry (UHPLC-QE Orbitrap MS) method (10). Our previous studies also indicated that the UHPLC-QE Orbitrap-MS/MS coupled with targeted and untargeted metabolomics analytical method is an effective approach for nutritional and phytochemical profiling of natural food and products (11, 12).

In the present study, a single batch of PE materials and its counterpart cofermented with *Citrus* peel were collected to investigate the non-volatile chemical profiles changes of PE during the process of Ganpu tea by an UHPLC-QE Orbitrap-MS/MS analysis method coupled with an integrated metabolomics strategy. The results would help to reveal their distinct chemical features of pu-erh tea after cofermented with *Citrus* peel.

## Materials and Methods

### Chemicals and Reagents

Liquid chromatography (LC)-grade methanol, acetonitrile, and formic acid were purchased from Merck (Darmstadt, Germany). Ultra-pure water (18.2 MΩ · cm) was prepared using a Milli-Q system (Millipore, Bedford, MA, USA). The reference standards of apigenin, kaempferol, luteolin, myricetin, quercetin, taxifolin, puerarin, vitexin, astragaline, hyperoside, isoquercitrin, kaempferol-3-O-rutinoside, rutin, gallic acid, epicatechin, (-)-gallocatechin, (-)-epigallocatechin, epigallocatechin gallate, (-)-gallocatechin gallate, (-)-epicatechin gallate, procyanidin B1, procyanidin B2, protocatechuic acid, trans-*p*-coumaric acid, caffeic acid, quinic acid (QA), chlorogenic acid, neochlorogenic acid, cryptochlorogenic acid, caffeine, theobromine, L-theanine, hesperidin, and the internal standard (IS) daidzein were purchased from Chengdu Push Bio-technology Co., Ltd. (Chengdu, China). The purities of the above references were higher than 98%.

### Preparation of Standard Solutions

Approximately 10 mg of each standard was accurately weighed and dissolved in methanol to obtain a stock solution at a concentration of 1 mg/mL.

### Sample Processing, Collection, and Pretreatment

Ganpu tea and PE samples were provided by Hongfeng Cooperative in Xinhui district of Guangdong province, China. Briefly, the pu-erh tea was cofermented with *Citrus reticulata* “Chachi” for 4 months in the circumstance of a temperature of 40°C and a humidity of 75%. After processing, the *Citrus* peel shells were removed and the pu-erh tea was used for analysis (*n* = 21). The PE samples treated at the same condition were used as the control group (*n* = 21). All samples were dried and ground using liquid nitrogen in a ceramic mortar with pestle.

### Preparation of Sample Solutions

All samples were dried and ground using liquid nitrogen in a ceramic mortar with pestle. Approximately 0.1 mg of each powered sample was accurately weighed, added with 5 μg internal standard, and then extracted with 10 mL methanol-water (70:30, v/v) in an ultrasonic bath (40 kHz, 250 W) for 30 min at room temperature. The extraction was centrifuged at 5,000 rpm for 15 min, and the supernatant was centrifuged at 17,500 rpm for 15 min. Then, 1 mL of supernatant was used for analysis. The quality control (QC) sample was made up by collecting 30 μL of every sample solution prepared previously. In the analysis batch, the QC sample was inserted in every six samples to monitor the reproducibility and stability of the method.

### UHPLC-Q-Orbitrap MS Analysis

The LC-MS experiments were performed on a U3000 UHPLC (Thermo Fisher, Waltham, MA, USA) coupled with a Q-Exacitve Orbitrap Plus hybrid MS system (Thermo Fisher Scientific, Rockford, IL, USA). The chromatographic separation was performed on a Waters HSS T3 column at a flow rate of 200 μL/min. The mobile phase consisted of acetonitrile (A) and 0.1% formic acid and the elution gradient was set as follows: 10% A (0–0.5 min), 10–40% (0.5–4 min), 40–55% A (4–10 min), 55–90% A (10–13 min), 90–10% A (13–15 min), 10% A (15–17 min). The column temperature was 25°C and the injection volume was 2 μL.

The MS data were acquired using Electron Spray Ionization (ESI) source both in negative and positive modes. The parameters were as follows: Ion spray voltage, 3,500 V in positive mode and 3,700 V in negative mode; capillary temperature, 350°C; aux gas, 15 arb; sheath gas, 40 arb; MS resolutions for survey scanning and data dependent acquisition (DDA) were 35,000 and 17,500, respectively; scan range, 130–1,300 m/z; and the normalized collision energy for DDA, 35 eV. Xcalibur software (version 3.1, Thermo Fisher, Waltham, MA, USA) was used for data acquisition.

### Qualitative Analysis

The identification procedure of the chemical components of PE could be described as the following three levels strategy: Level 1, directly and accurately identified by comparing the retention time, quasi-molecular and fragment ions with reference standards analyzed under the same condition; Level 2, identified by comparing the determined molecular formula and characteristic fragment ions in their MS/MS spectra to the data of known compounds that have already been reported in PE; Level 3, identification was conducted based on the fragmentation patterns and diagnostic fragment ions and/or neutral losses of reference that summarized from the analysis of standards and known compounds.

### Quantitative Analysis

Among all the identified components pool, 89 components that showed high peak intensities, good peak shape and separation, were selected for quantification. Since only 29 standards were used for quantification, the quantity of these components was directly calculated by the calibration curves, while the left components were calculated by using the standard curves with a similar structure to that of the target component. For example, the quantification of gallic acid derivatives was determined by the standard curve of gallic acid; all of apigenin-glycosides by vitexin; 6-Carboxyl-(-)-Gallocatechin by (-)-Gallocatechin; and caffeic acid derivatives by caffeic acid. As to some compounds belonging to a set of isomers that showed identical MS behavior and similar retention time, their contents were calculated combinedly.

The quantification method was validated for linearity, limit of detection (LOD), limit of quantification (LOQ), inter-day, and intraday precision of 29 reference compounds. The calibration curves were obtained by plotting the peak areas vs. concentrations. The LOD and LOQ were determined by the signal-to-noise ratio of more than 3 and of 10, respectively. Samples at three different levels (low, middle, and high) were prepared and injected six times within a single day and duplicate for three consecutive days to evaluate the intraday and inter-day precision, respectively, which were then calculated and expressed as relative standard deviation (RSD%).

### Data Preprocessing and Statistical Analysis

The untargeted metabolomics analysis was conducted using XCMS online software (www.xcmsonline.scripps.edu/) to preprocess the raw files (parameters were set as follows: ppm, 10; prefilterK, 10; prefilterl, 200,000) with a final table of MS peaks included information of accurate molecular weight (*m/z*), retention time, and peak area. The data were then normalized using IS and exported into SIMCA-P 14.1 (Umetrics, Sweden) for unsupervised principal component analysis (PCA) and hierarchical cluster analysis (HCA).

The quantitative data of compounds were imported into Statistical Product and Service Solution (SPSS) version 19.0 (IBM, USA) for an Independent Samples *T*-test, which was performed to analyze the statistical difference between two groups. The compounds displayed with significant difference (*P* < 0.05) were retained for further orthogonal partial least squares discriminant analysis (OPLS-DA). *R*^2^ and *Q*^2^ values were used to evaluate the goodness and predictability of the OPLS-DA model and the 200 times permutation tests to evaluate whether the model was overfitting. Then, variable influences in projection (VIP) were generated from S-plot and those compounds with VIP>1 and fold change (FC) > 1.8 were selected as the potential chemical markers. The predictive ability of these markers was further evaluated by the receiver operating characteristic curve (ROC) and the area under curve (AUC) values, which were performed by using SPSS. Heatmap analysis was conducted using Pheatmap package in RStudio environment to display the quantitative changes of the chemical constituents more intuitively.

## Results and Discussions

**Figure 2:**
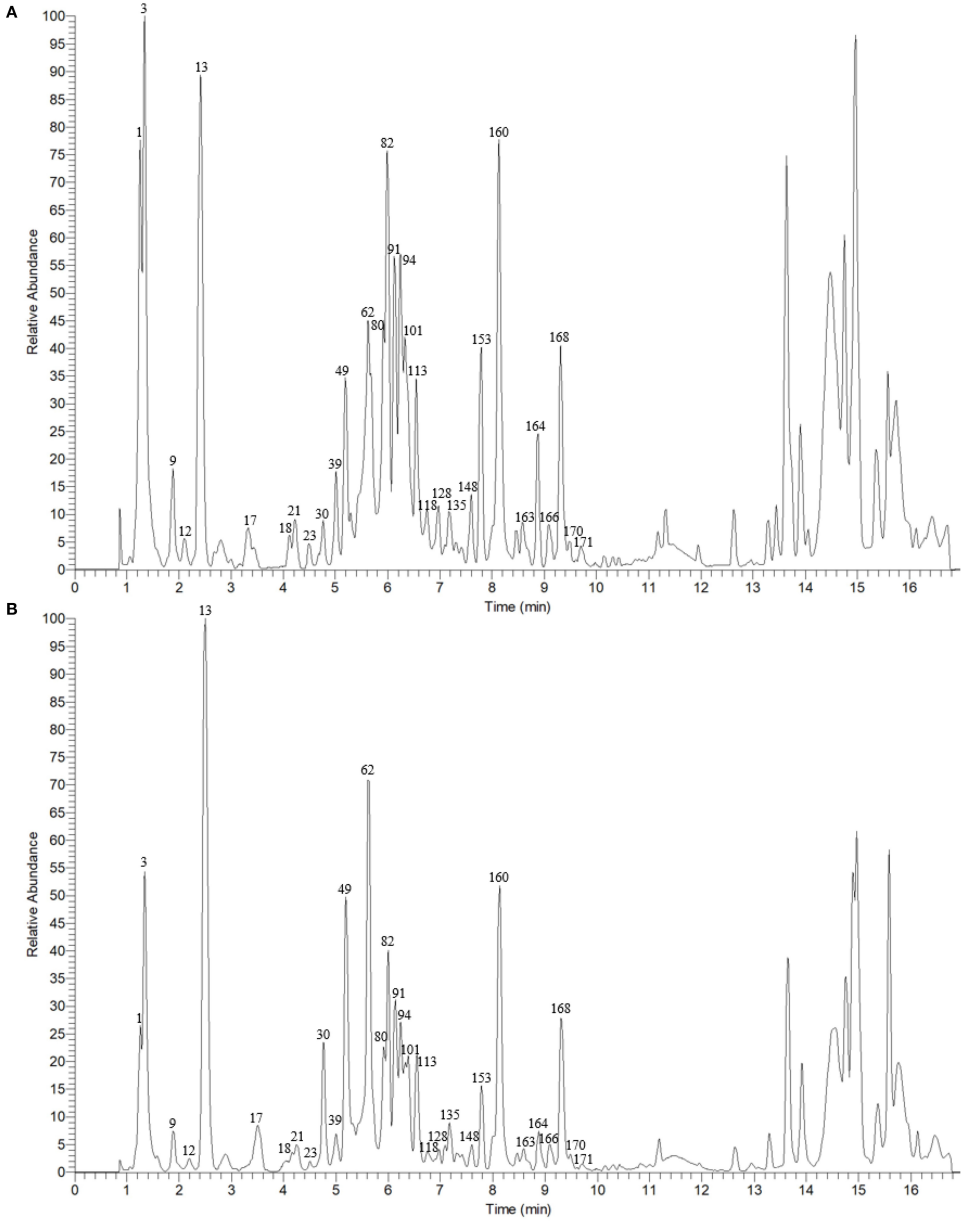
The total ion chromatograms (TICs) of Ganpu tea **(A)** and Pu-erh tea **(B)** in negative ion mode.

The typical total ion chromatograms (TICs) of Ganpu tea and PE were illustrated in Figure 2, where the disparity in the relative peak height and peak area can be concluded by visual inspection. In order to obtain a more detailed knowledge concerning the changes of the chemical constituents, the chemical investigation was conducted first.

### Identification of Chemical Components

Based on the qualitative analysis strategy, a total of 171 compounds were identified or tentatively characterized, including phenolic acids, flavan-3-ols, flavonoids/flavonoid glycosides, and others, 31 of them were confidently identified according to the standards (level 1), 94 were tentatively identified according to literature data (level 2), and 45 of them were putatively identified as potential new compounds based on level 3 (27 flavonoids, 11 flavan-3-ols, and seven phenolic acids) (Supplementary Table 3).

The main flavonoids in PE were quercetin, kaempferol, myricetin, and their glycosides. Several of them were identified by comparing accurate molecular mass and retention time to those reference standards based on level 1. The other chemical components were identified based on the accurate molecular mass and their characteristic MS fragments by level 2/3. Flavonoid glycosides include O-glycosides and C-glycosides, which can be differentiated by the distinct fragmentation patterns in QE Orbitrap MS. The characteristic fragmentation pattern of flavonoid C-glycosides was featured by the interior cleavage of glycoside, which produce several [M–n×CH_2_O] fragment ions such as hexose neutral loss C_4_H_8_O_4_ or C_3_H_6_O_3_, pentose neutral loss C_3_H_6_O_3_ or C_2_H_4_O_2_) (13). As for flavonoid O-glycosides, the MS fragmentation was featured by producing the characteristic base fragment ion of [M–glycosides] such as the neutral loss of C_6_H_10_O_5_ (162, hexose), C_6_H_10_O_4_ (146, deoxyhexose), and C_5_H_8_O_4_ (132, pentose) (14). The aglycone structure can further lose ${\text{CH}}_{3}^{\cdot }$ and CO_2_ or display the retro–Diels–Alder (RDA) cleavage of the C ring. Take compound 109 for example, it showed a [M–H]^−^ at *m/z* 599.1044 (C_28_H_23_O_15_), which further produced the fragment ions of [M–galloyl]^−^ at *m/z* 447.0933 (C_21_H_19_O_11_) and [GA–H]^−^ at *m/z* 169.0132 (C_7_H_5_O_5_), indicating a gallic acid moiety bind to the structure (herein GA denotes gallic acid). MS^2^ at *m/z* 447.0933 further fragmented into the base fragment ion at *m/z* 285.0401 (C_15_H_9_O_6_) due to loss of 162 Da (C_6_H_10_O_5_) of hexose (hex). By referring to the literature data (15), it was identified as kaempferol-7-(6″-galloylglucoside). Compounds 126, 132, 142, and 147 all showed the common characteristic fragment ions at *m/z* 285.0403 (C_15_H_9_O_6_) and *m/z* 163.0390 (C_9_H_7_O_3_), which represented the characteristic fragment ions of kaempferol and coumaric acid (Co) moieties, respectively. According to the determined elemental compositions, 126, 132, 142, and 147 could be tentatively identified as kaempferol-O-coumaric acid-hexose-di-deoxyhexose, kaempferol-O-coumaric acid-pentose-deoxyhexose-hexose, kaempferol-O-coumaric-deoxyhexose-hexose, and kaempferol-O-coumaric acid-hexose, which were detected in PE for the first time. For compounds 42, 83, 119, and 129, they exhibited a series of fragment ions of [M–n×CH_2_O], such as [M−6×CH_2_O] at *m/z* 415.1034, [M−7×CH_2_O] *m/z* at 385.0924, [M−8×CH_2_O] at *m/z* 355.0817 for compound 42, the [M−3×CH_2_O] at *m/z* 343.0818, [M−4×CH_2_O] at *m/z* 313.0717 for compound 83, the [M−2×CH_2_O] at *m/z* 357.0610, [M−3×CH_2_O] *m/z* 327.0511 for compound 119, and [M−2×CH_2_O] at *m/z* 341.0661, [M−3×CH_2_O] at *m/z* 311.0559 for compound 129. Furthermore, these compounds showed a distinct fragment ion at *m/z* 271.0612 (C_15_H_11_O_5_, naringenin) or *m/z* 285.0340 (C_15_H_9_O_6_, kaempferol) due to the loss of glycosides. Thus, they were putatively identified as naringenin-C-di-hexose, naringenin-C-hexose, kaempferol-C-pentose, and kaempferol-C-deoxyhexose, among them, compounds 41 and 119 were detected in PE for the first time.

**Figure 3:**
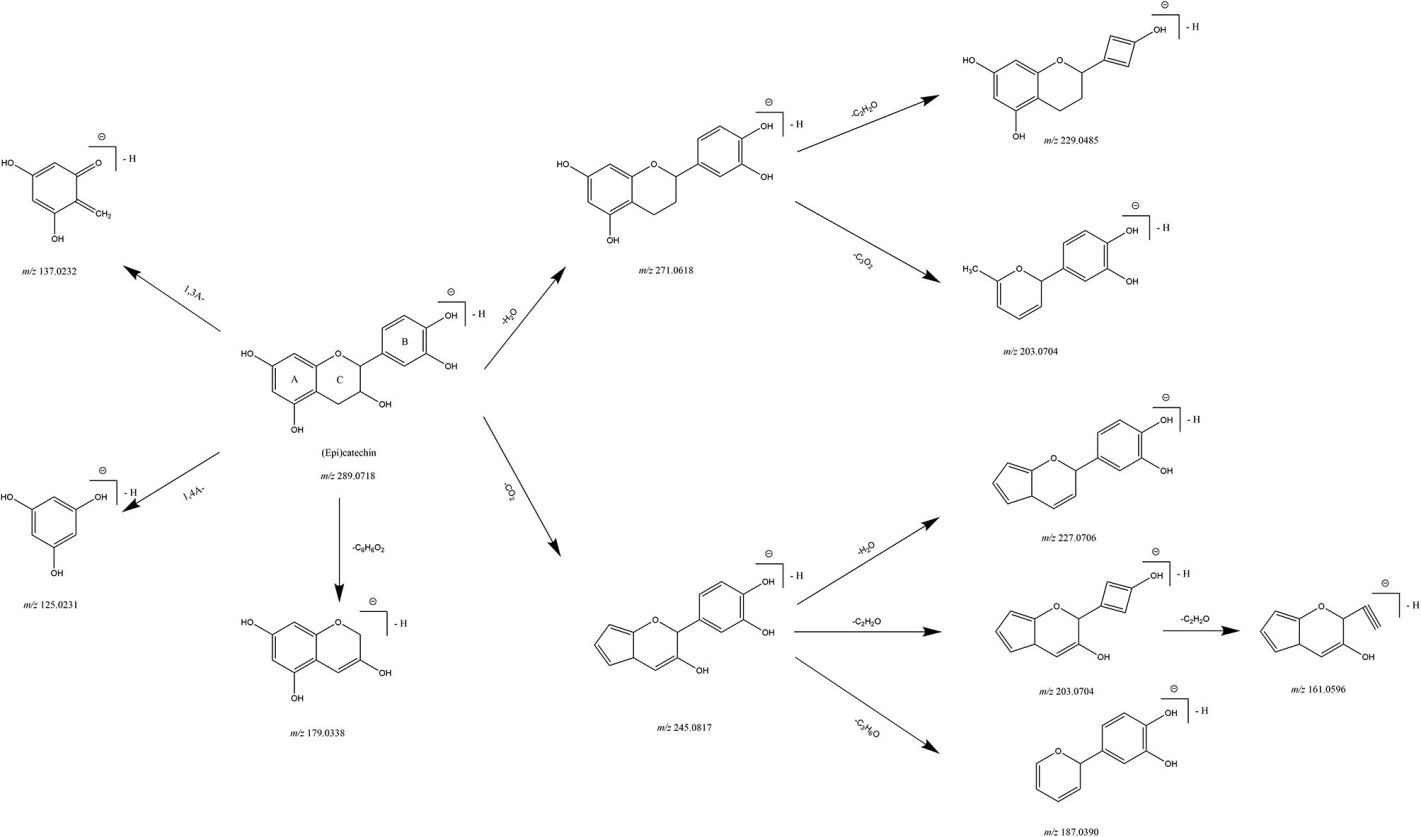
Proposed fragmentation pathway of Catechin/Epicatechin.

The major flavonols in PE were catechin/gallocatechin and its dimers or gallate derivatives, which can be featured by the characteristic fragment ions at *m/z* 289.0720, *m/z* 305.0666, and *m/z* 169.0132. The characteristic MS fragmentation rule of catechin was summarized by analyzing the reference standard (Figure 3). First, cleavage of C ring resulting the ^1, 3^A, ^1, 4^A fragments at *m/z* 137.0230, *m/z* 125.0231. Second, loss of B ring (C_6_H_6_O_2_) producing the characteristic fragment ion at *m/z* 179.0341. In addition, loss of H_2_O can be followed by a further loss of B ring (C_2_H_2_O) and A ring (C_3_O_2_). Similarly, after losing of CO_2_ through A ring at *m/z* 245.0801, it further produced a series of product ions that loss H_2_O, C_2_H_2_O, or C_3_H_6_O fragments. For example, compound 32 had a quasi-molecular ion at *m/z* 593.1299 and fragment ions at *m/z* 305.0667 and *m/z* 289.0723. Therefore, the structure can be described as a dimer of catechin and gallocatechin. Combined with literature data (16), compound 32 was identified as (epi)gallocatechin–(epi)catechin isomer. The [M–H]^−^ ions of compounds 15 and 38 were at *m/z* 481.0993 and *m/z* 465.1040, and the characteristic fragment ions of them were at *m/z* 305.0664 (C_15_H_13_O_7_) and *m/z* 289.0719 (C_15_H_13_O_6_), respectively, due to the loss of 176 Da, referring to a loss of glucuronic acid (glc ua) with other common fragment ions of [M–CO_2_-2×CH_2_O], and [M–CO_2_-3×CH_2_O]. Thus, compounds 15 and 38 were tentatively identified as (-)-gallocatechin-glucuronic acid and catechin-glucuronic acid, which were detected from PE for the first time. The prominent fragment ions of compound 71 at *m/z* 289.0722 and *m/z* 169.0132 were referring to the quasi-molecular ions of catechin and gallic acid respectively, combined with the other fragment ions of [M–galloyl–CO_2_-2×CH_2_O] and [M–galloyl–CO_2_-3×CH_2_O]. Thus, compound 71 could be tentatively identified as catechin gallate-glucuronic acid. Compounds 26 and 85 were putatively identified as catechin -C-hexose and catechin-C-deoxyhexose-hexose according to the fragment ions of [M–n×CH_2_O] generated from a series of interior cleavages of glycosides and the characteristic fragment ion at *m/z* 289.0714 (quasi-molecular ions of catechin). Compound 27, which showed ion at *m/z* 331.0819, fragmented into characteristic ion at *m/z* 289.0719 (C_15_H_13_O_6_) due to a neutral loss of 42 Da (acetyl residue), so it was identified as acetylated (epi)catechin. Compounds 15, 27, 38, 71, and 85 were detected from PE for the first time.

For phenolic acids, the fragmentation rules can be simply expressed as the loss of H_2_O and CO_2_, which leads to the production of fragment ions of [M–H_2_O]^−^ and [M–CO_2_]^−^. Most phenolic acids that acylate with QA were featured by producing the common characteristic fragment ion at *m/z* 191.0552 and the basic ion peak of the corresponding phenolic acid like gallic acid (*m/z* 169.0132), caffeic acid (*m/z* 179.0340), and trans-*p*-Coumaric acid (*m/z* 163.0391). Based on this, compounds 12, 39, and 41 were identified as theogallin, *p*-coumaroylquinic acid, and chlorogenic acid, respectively. Several phenolic acids were also glycosylated. Compounds 1, 7, and 90 showed [M–H]^−^ at *m/z* 353.1086 (C_13_H_21_O_11_), *m/z* 477.1255 (C_19_H_25_O_14_), and *m/z* 341.0907 (C_15_H_17_O_9_), respectively, both of compounds 1 and 90 shared a similar loss of 162 Da (hex) and generated the prominent ions at *m/z* 191.0552 (QA) and *m/z* 179.0348 (caffeic acid), respectively, while compound 7 produced the prominent ion at *m/z* 169.0132 (GA) due to the loss of 308 Da (rutinoside). Furthermore, compound 90 produced fragment ions of [M−4×CH_2_O] and [M−5×CH_2_O], which indicated a C-glycoside binding to the caffeic acid. Thus, compounds 1, 7, and 90 were tentatively identified as QA-O-hexose, gallic acid-O-rutinoside, and caffeic acid-C-hexose, respectively, which were detected in PE for the first time.

### Untargeted Metabolic Profiling of Ganpu Tea and PE

**Figure 4:**
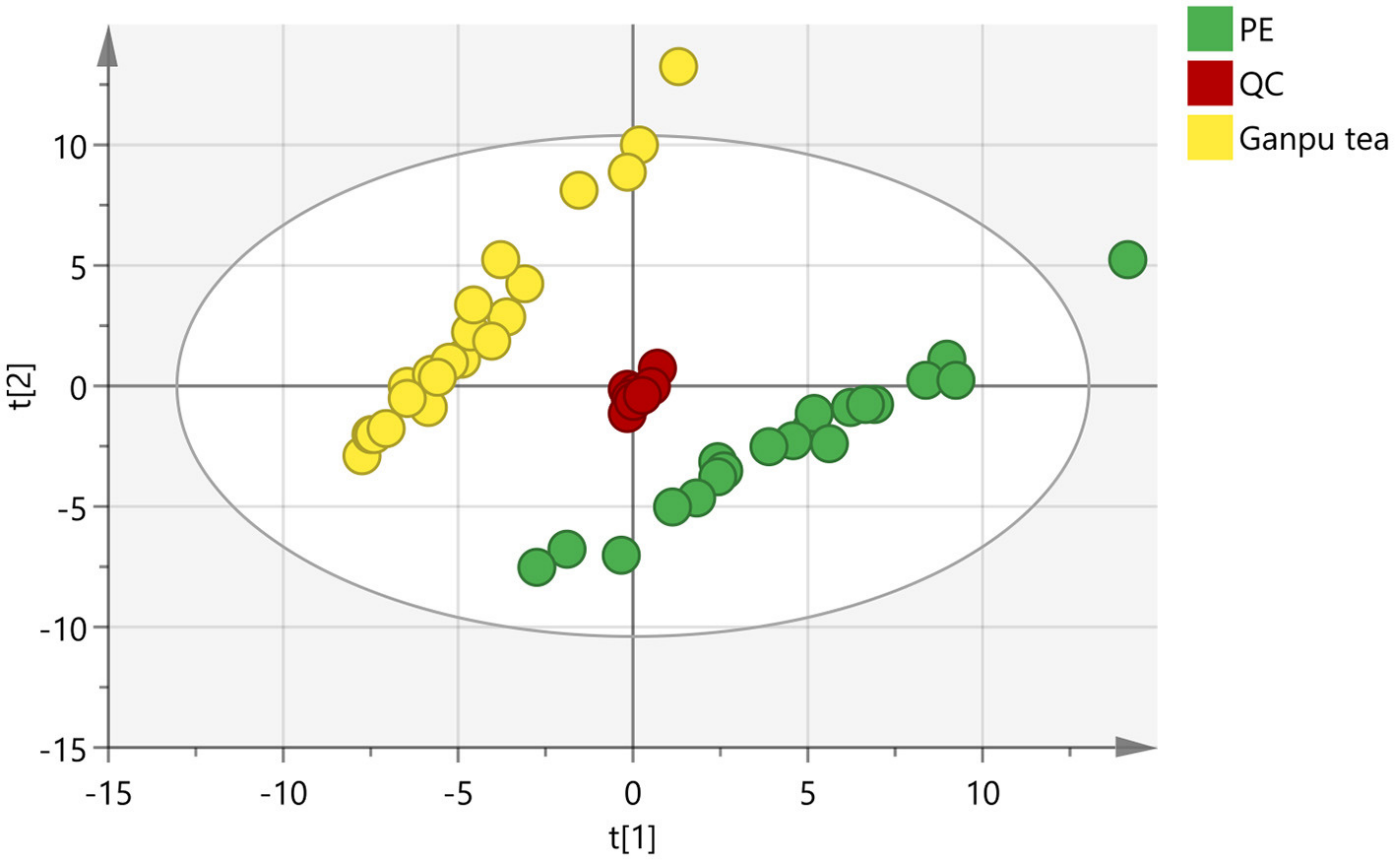
Score plot of principle component analysis of 2017 features (*R*^2^X = 0.834, *Q*^2^ = 0.741). Green circles represent Pu-erh tea (PE) samples, yellow circles represent Ganpu tea (Pu-erh tea that cofermented with *Citrus* peel) samples, and red circles represent quality control (QC) samples.

**Figure 5:**
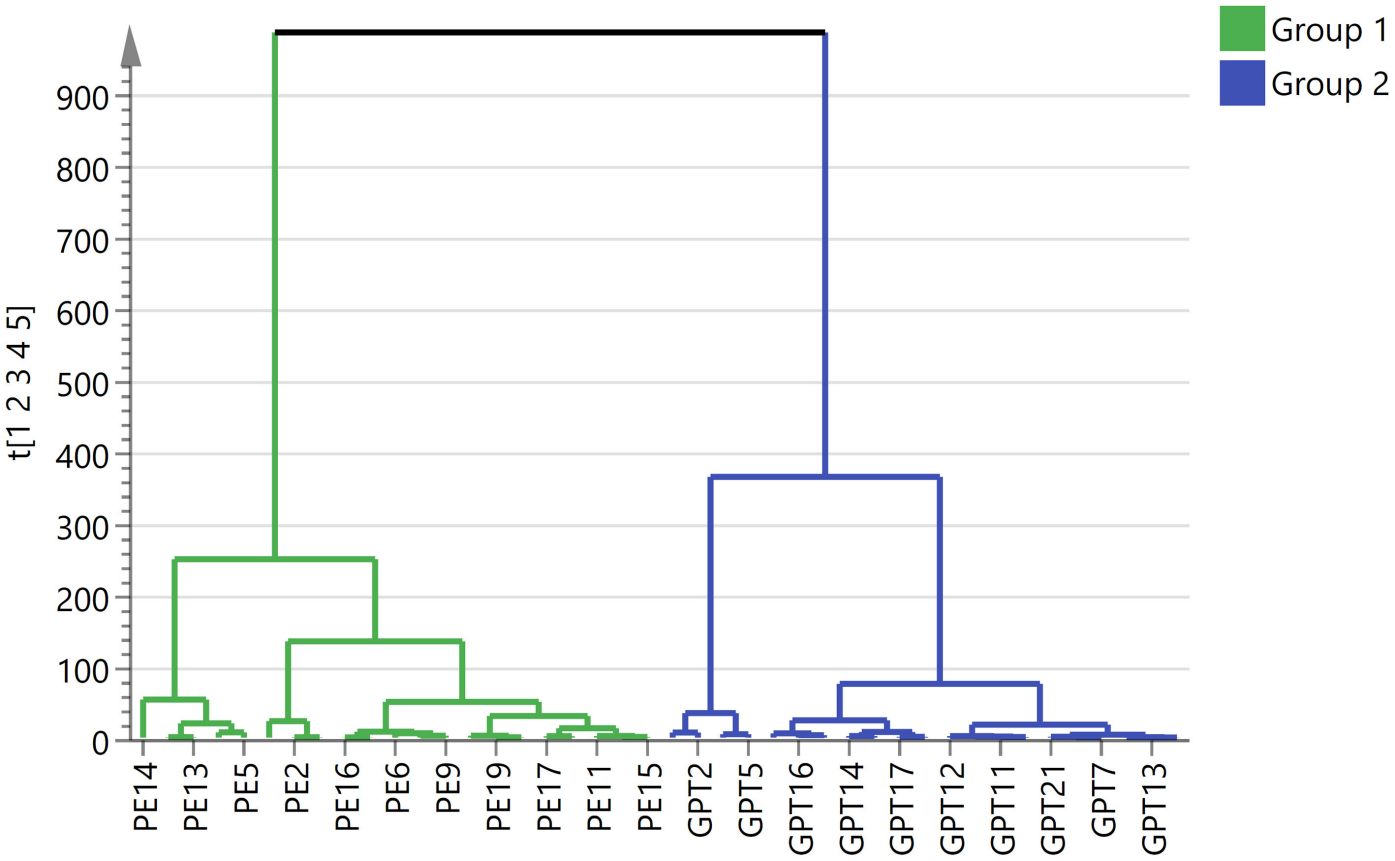
Clustering analysis of 42 tea samples. Group 1 in green represents 21 Pu-erh tea samples and Group 2 in blue represents 21 Ganpu tea samples.

In order to obtain and compare the metabolic profiles of PE and Ganpu tea, multivariate analysis was performed by using an untargeted metabolomics method. The quality evaluation of the method was achieved by calculating the RSD% of all the QC sample injections during the analysis batch. Statistical analysis of RSD of all 2017 features from the QC samples showed that features with RSD% <5% account for 34.16%, <20% for 89.24% and between 20 and 30% for 10.76%, which demonstrated the good reproducibility and stability of the method (Supplementary Figure 1A). The unsupervised PCA and HCA models were established to obtain an overview of the differences in tea samples and determine whether the chemical profile of PE tea was influenced by the cofermentation with *Citrus* peel. The unsupervised PCA score plot was shown in Figure 4, which displayed a good separation between two tea groups, while the QC group was clearly clustered in the origin of orthogonal coordinate, which indicated a good predictability and repeatability of the model. From the HCA plot (Figure 5), the 42 samples were explicitly divided into two clusters; the first cluster included 21 PE samples and the second included 21 Ganpu tea samples. According to the results of PCA and HCA, the PE fermented with the *Citrus* was obviously different from the ordinary PE in chemical composition, indicating a strong correlation between chemical metabolism of PE and cofermentation process.

### Quantitative Comparison and Multivariate Analysis

The validation of the established quantitative method in terms of linearity, LOD, LOQ, inter-day, and intraday precision was conducted by using 29 standards, which covered the main flavonoids, flavone glycosides, catechins, phenolic acids, and alkaloids in tea. As shown in Table 1, all the 29 standards showed good linearities between concentrations and corresponding MS peak area with correlation coefficients >0.996. The intraday and inter-day RSDs of most standards at three different levels were <10%, which displayed good precision of the method. The LODs and LOQs of analytes indicated the good sensitivity of the quantitative method with the results of 0.2–1.0 and 0.4–4.0 ng/mL, respectively.

**Table 1:** Validation results of the quantitative method.

	**Standard**	**Linear range (μg/mL)**	**Linear equation**	***R* ^**2**^**	**LOD (ng/mL)**	**LOQ (ng/mL)**	**Precision (RSD, %)**	
							**Intraday (low, middle, high)**	**Inter-day (low, middle, high)**
1	Gallic acid	0.0625-64	y = 104867528.0426x + 63531869.8110	0.9974	0.4	1.0	2.67,1.87,2.41	9.14,2.23,2.42
2	Quinic acid	0.015625-16	y = 233490533.2248x + 18924142.9056	0.9985	1.0	4.0	4.84,1.56,2.17	9.87,2.80,2.49
3	Epicatechin	0.015625-16	y = 272585339.2833x + 51325033.6099	0.9969	0.2	0.6	2.62,1.42,3.37	8.84,4.39,2.20
4	Hyperoside	0.015625-8	y = 336131315.6349x + 26185263.8044	0.9970	0.6	1.0	2.40,4.52,4.39	5.14,7.94,6.83
5	Chlorogenic acid	0.011719-12	y = 236822254.5812x-8681388.1304	0.9997	0.8	2.0	0.90,1.48,0.99	6.11,2.09,2.23
6	(-)-Gallocatechin	0.007813-8	y = 181374708.3263x-7335710.7997	0.9995	0.6	1.0	1.97,1.10,0.68	7.34,2. 13,1.88
7	Epigallocatechin	0.007813-8	y = 234652428.5065x + 6820882.4056	0.9983	0.6	1.0	2.37,1.53,1.72	13.31,3.61,1.19
8	Procyanidin B2	0.007813-8	y = 207039382.9164x-3732613.5846	0.9998	0.6	1.0	1.88,1.31,2.10	5.87,2.04,2.08
9	Neochlorogenic acid	0.007813-8	y = 233286007.3678x-2865114.2856	0.9998	1.0	4.0	1.65,1.25,2.10	5.35,1.32,2.06
10	Epigallocatechin gallate	0.007813-8	y = 201974972.0708x + 1577496.8116	0.9979	0.8	2.0	1.77,2.40,3.10	9.27,1.61,3.27
11	Rutin	0.007813-8	y = 309573862.9222x + 5346801.5931	0.9990	0.6	1.0	3.41,6.80,6.85	4.26,4.51,9.38
12	trans-p-Coumaric acid	0.03125-2	y = 253915567.7584x + 2156877.9752	0.9998	0.6	2.0	3.38,12.22,14.65	6.85,15.48,11.37
13	Quercetin	0.007813-8	y = 693579297.3999x + 44321253.9048	0.9983	0.6	1.0	5.74,8.09,3.08	10.30,4.42,7.17
14	Kaempferol-3- O-rutinoside	0.007813-8	y = 317275235.2128x + 11376296.5761	0.9996	0.2	0.6	5.24,5.84,9.94	8.53,11.74,4.47
15	Astragalin	0.007813-8	y = 440871245.5237x + 16893068.9826	0.9990	0.2	0.4	3.22,7.27,2.36	9.99,4.76,3.52
16	Protocatechuic acid	0.007813-8	y = 133443163.8279x + 5417038.8257	0.9987	1.0	2.0	1.33,1.71,0.77	6.43,2.17,1.75
17	Kaempferol	0.003906-4	y = 1145742735.5217x + 10566558.1343	0.9989	0.8	1.0	2.86,3.78,7.53	9.95,5.74,4.62
18	(-)-Gallocatechin 3-O-gallate	0.003906-4	y = 247957157.35x-4760951.20	0.9998	0.8	1.0	3.82,1.50,3.41	6.17,4.47,3.49
19	Vitexin	0.003906-4	y = 354134913.2230x + 134415.2330	0.9999	0.4	1.0	2.66,7.22,11.12	3.96,4.64,4.71
20	Myricetin	0.007813-2	y = 595502169.4122x-12890424.3678	0.9994	0.8	1.0	2.49,4.65,4.40	11.86,7.45,5.88
21	Procyanidin B1	0.001953-2	y = 141557727.2076x-2136920.2090	0.9995	0.6	1.0	3.79,2.36,3.04	3.87,1.70,2.71
22	Caffeic acid	0.000977-0.25	y = 290483148.0039x + 2032122.7323	0.9971	0.6	2.0	3.77,4.32,5.97	6.14,4.91,5.73
23	Taxifolin	0.001953-0.5	y = 475841384.5211x-247779.2512	0.9997	0.4	0.6	5.61,4.76,3.55	8.39,7.22,9.29
24	Luteolin	0.001953-0.5	y = 1185045545.0720x-3667626.1579	0.9998	0.4	1.0	4.44,5.49,6.16	14.05,7.28,5.64
25	Apigenin	0.000488-0.5	y = 1504164432.8718x-879823.2686	0.9997	0.4	0.8	4.92,6.32,5.41	9.27,6.72,5.47
26	Epicatechin gallate	0.015625-8	y = 306041466.8868x + 16782555.2605	0.9988	0.6	1.0	5.93,5.95,5.90	8.94,8.27,5.15
27	Theobromine	0.03125-32	y = 306823878.3108x + 50508654.8548	0.9989	0.1	0.4	2.14,2.87,0.95	2.07,2.46,3.31
28	Caffeine	0.03125-32	y = 523214802.6364x + 265990080.5366	0.9981	0.1	0.4	1.02,3.08,1.00	1.45,1.30,3.43
29	L-Theanine	0.001953-2	y = 258253217.3246x – 7060817.7917	0.9978	0.4	2.0	0.55,2.56,3.21	1.62,2.95,5.19

**Figure 6:**
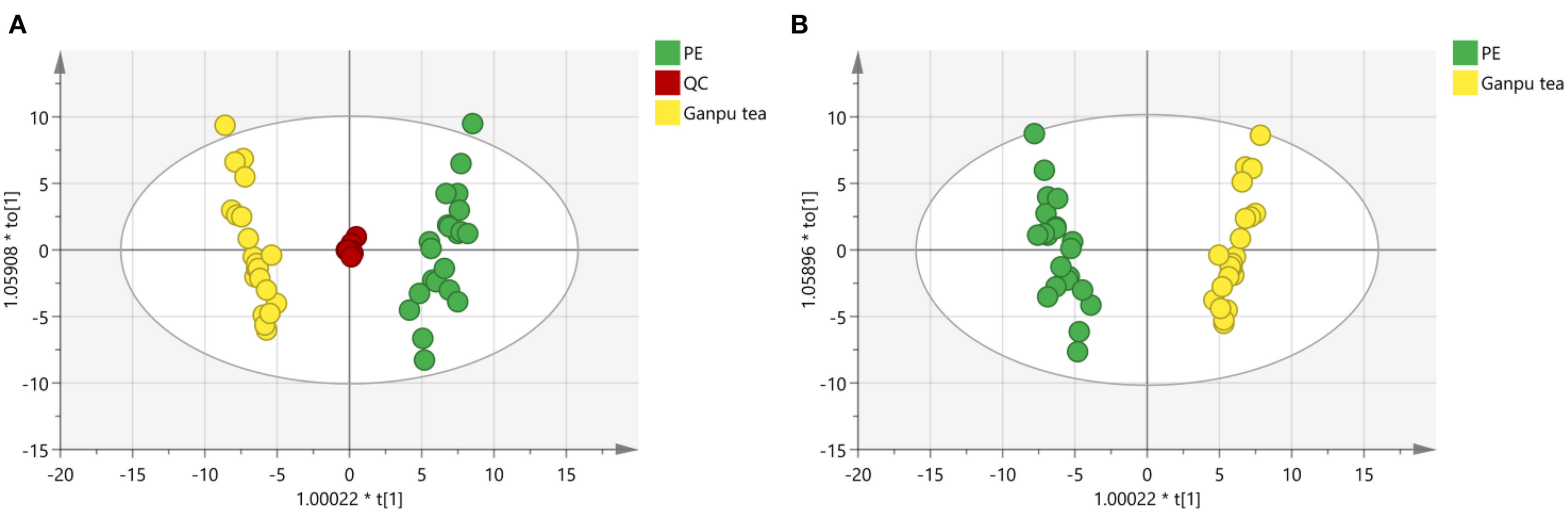
OPLS-DA analysis of 67 features: **(A)** Score plot of orthogonal partial least squares analysis of PE, Ganpu tea, and QC samples (*R*^2^X = 0.805, *R*^2^Y = 0.560, *Q*^2^ = 0.556). **(B)** Score plot of orthogonal partial least squares analysis between PE and Ganpu tea (*R*^2^X = 0.809, *R*^2^Y = 0.975, *Q*^2^ = 0.970). Green circles represent Pu-erh tea samples, yellow circles represent Ganpu tea samples, and red circles represent quality control (QC) samples.

**Figure 7:**
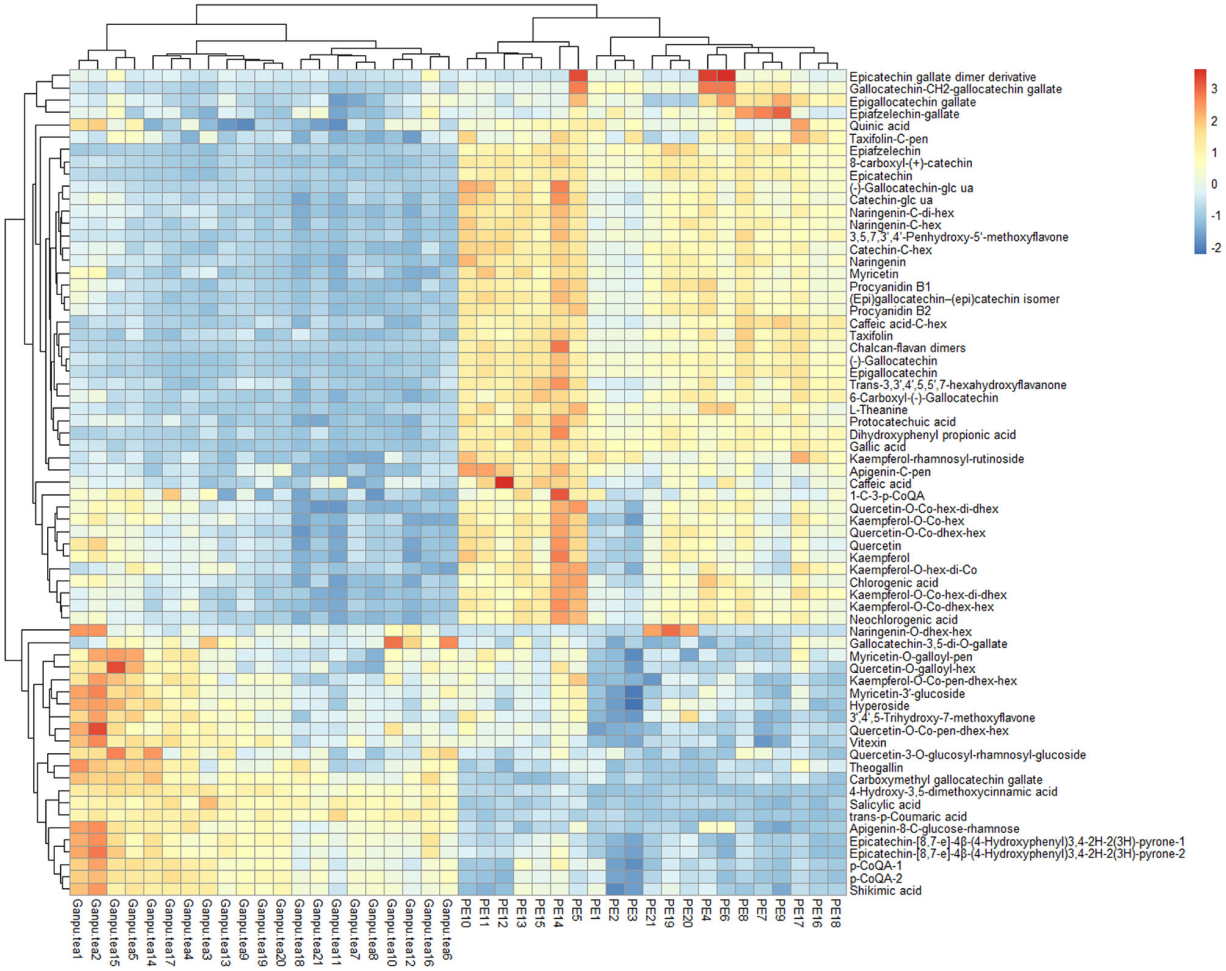
Heatmap constructed by the quantitative data of 67 features. Heatmap shows that Pu-erh tea and Ganpu tea have been well-distinguished.

**Figure 8:**
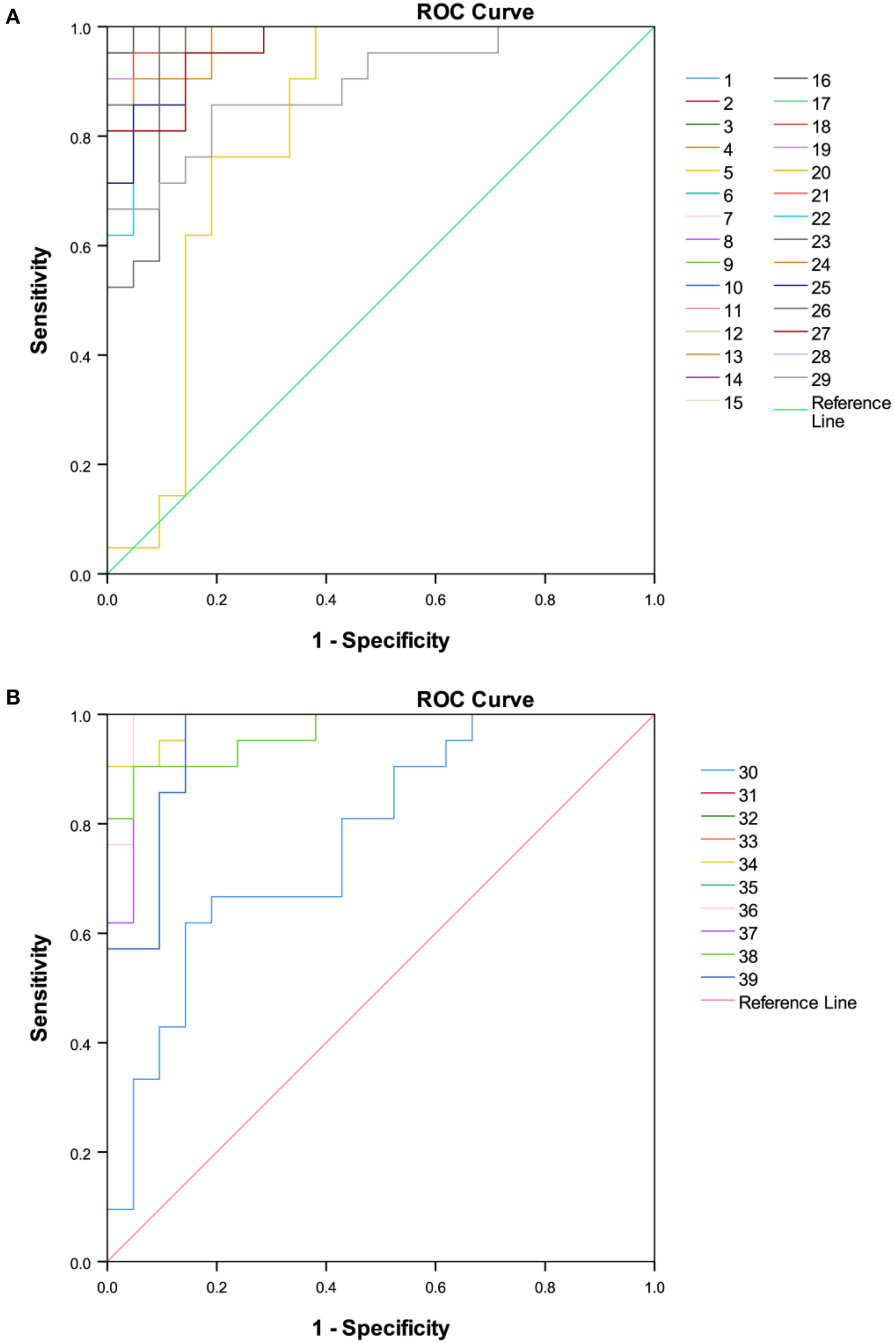
Receiver operating characteristic curve (ROC) of 39 potential chemical markers selected by variable influences in projection (VIP) values. **(A)** Features with higher quantity level in PE. **(B)** Features with higher quantity level in Ganpu tea.

A total of 89 identified components were selected for quantitative analysis using the quantitative method established above. The results showed that the total content of flavonoid glycosides was basically unchanged and the aglycones with slightly decreasing, whereas the total content of phenolic acids and flavan-3-ols were dropped sharply in the Ganpu tea samples. The univariate analysis of 89 targets showed that 67 of them were displayed with significant differences (Supplementary Table 1), which were then adopted for further multivariate statistical analysis. As shown in Figure 6, the PCA **(Figure 6A)** and OPLS-DA **(Figure 6B)** score plot displayed two clear clusters of PE and Ganpu tea, respectively. The relevant *R*^2^Y = 0.975 and *Q*^2^ = 0.970 significantly indicated the validity and good predictability of the model. The 200 iterations permutation test (Supplementary Figure 2) indicated that there was no overfitting of the model. A detailed content differences of each target between the two groups were illustrated in a heatmap (Figure 7), where samples were displayed on the horizontal axis, compounds were on the vertical axis, and content differences were reflected by the color (color from blue to red indicated constituent content from low to high). The contents of most flavan-3-ols (epicatechin, epigallocatechin, (-)-gallocatechin, chalcan-flavan dimers, procyanidin B2, and so on), most major flavonoid aglycones (naringenin, myricetin, quercetin, kaempferol), the major phenolic acids (gallic acid, neochlorogenic acid, chlorogenic acid, caffeic acid, QA), two naringenin/quercetin glycosides, most of kaempferol glycosides, and L-theanine were decreased after cofermented with *Citrus* peel, which were showed at cluster 1 of targets with a higher content in PE. Whereas, the compounds with higher content in Ganpu tea were automatically gathered into cluster 2 in the following rows, which included gallocatechin-3,5-di-O-gallate, carboxymethyl gallocatechin gallate, epicatechin-[8,7-e]-4β-(4-Hydroxyphenyl)3,4-2H-2(3H)-pyrone, trans-p-Coumaric acid, p-Coumaroylquinic acid, theogallin, and most of glycosides (vitexin, apigenin-8-C-glucose-rhamnose, two myricetin glycosides, four quercetin glycosides, and so on). The characteristic compounds that contribute greatly in classifying tea samples can be initially screened based on the VIP value from S-plot constructed by OPLS-DA model and FC, 16 compounds with VIP value > 1 and FC value > 1.8 were selected (Supplementary Table 2). ROC analysis was then performed to verify the correct predictability of those characteristic compounds (Figure 8), AUC values showed that 13 of the 16 compounds selected by the value of VIP and FC were equal to 1 (epicatechin, epigallocatechin, (-)-gallocatechin, 8-carboxyl-(+)-catechin, epiafzelechin, dihydroxyphenyl propionic acid, taxifolin, chalcan-flavan dimers, catechin-C-hexose, L-Theanine, (-)-gallocatechin-glucuronic acid, trans-*p*-coumaric acid, 4-hydroxy-3,5-dimethoxycinnamic acid) (Supplementary Table 2), which indicated an excellent capability of the selected chemical markers to distinguish PE and Ganpu tea.

Polyphenols are the main secondary metabolites in tea leaves, which account for 18–36% of the tea dry weight. The most critical biosynthetic pathway of tea plant include shikimic acid pathway, phenylpropanoid pathway, and flavonoid pathway (17). During the cofermentation process with *Citrus*, the chemical composition of pu-erh tea varied obviously in types and quantities. According to the quantitative results, the total flavonoid glycosides contents in pu-erh tea were displayed with no significant change after cofermented with *Citrus*, whereas the total contents of flavonoid aglycones, phenolic acids, and flavan-3-ols were significantly decreased. By referring to the metabolic pathway of polyphenols biosynthesis in tea plants (Supplementary Figure 3), the effects of *Citrus*-tea cofermentation on the contents of major constituents in pu-erh tea could be described as follows: the major flavonoid aglycones such as naringenin, apigenin, kaempferol, myricetin, and quercetin were all significantly decreased in pu-erh tea after the cofermentation process. Several major flavonoid glycosides including vitexin and apigenin-8-C-glucose-rhamnose, most of the quercetin glycosides and myricetin glycosides were significantly increased, which might be related to the cofermentation process with *Citrus* that is rich in glycosides. Most literature has reported that the gallic acid was elevated due to the degradation of polyphenols during the fermentation process of PE (18). However, in this study, the content of gallic acid was significantly decreased (19), corresponding to a literature that reported that gallic acid content was decreased after long-term pile-fermentation, although the mechanism has not been revealed yet. In addition, according to the 16 selected potential chemical markers, 11 of them belong to flavan-3-ols type with a decreased trend after cofermentation process. As flavan-3-ols were considered to be related to the bitterness and astringency of tea, we can speculate that the *Citrus*-tea cofermentation process has accelerated the fermentation of PE and the significantly decreasing trend of major flavan-3-ols may contribute to the unique flavor of Ganpu tea. Interestingly, hesperidin, a representative compound in *Citrus* peel, was first found in pu-erh tea after cofermented with *Citrus*, the result of which may be due to a chemical transformation or the contaminate effect of *Citrus* peel on pu-erh tea during the *Citrus*-tea cofermentation process. Hesperidin possesses a variety of biological activities, such as anti-inflammatory and hypolipidemic (20, 21), and it has been reported that the solubility of hesperidin could be enhanced by a fermented tea extract containing polyphenols (22), thus, it could be inferred that the hesperidin newly found in pu-erh tea was expected to further contribute to the health effect.

Comprehensive analysis showed that the expression of phenolic acids, flavan-3-ols, and flavonoid/flavonoid glycosides in pu-erh tea varied significantly after cofermented with *Citrus*. On the one hand, most of the main flavan-3-ols, phenolic acids, and flavonoids were decreased, which are consistent with the previous study reports that almost all of the main secondary metabolites of the tea plant were degraded after pile-fermentation (19, 23). And the result of decreasing trend of gallic acid content was consistent with the long-term pile-fermentation effect of PE. Thus, it can be inferred that the cofermentation process with *Citrus* accelerated the change of chemical transformation of pu-erh tea such as the degradation and oxidative polymerization of catechin. On the other hand, several flavonoid glycosides have increased. Because of the high contents of flavonoids in *Citrus* peel, we speculated that the process of cofermentation with *Citrus* promoted the conversion of flavonoids to flavonoid glycosides, which might result from multiple enzymatic effects. Furthermore, catechins and flavones were the major compounds contributing to the health benefit of pu-erh tea, as well as the bitterness and astringency of tea infusion (24). It has been reported that these two kinds of components decreased significantly after the manufacturing of postfermentation, which was attributed to a series of reactions during microbial fermentation, including degradation, oxidation, condensation, structural modification, methylation, and glycosylation (24, 25). In addition, the poor bioavailability of catechins and the low oral threshold of flavones have been widely recognized (26, 27). In this work, the contents of catechins and flavones were further obviously reduced after cofermented with *Citrus*, structural modification transformation products such as the flavonoid glycosides, carboxymethyl gallocatechin gallate, and epicatechin-[8,7-e]-4β-(4-Hydroxyphenyl)3,4-2H-2(3H)-pyrone were significantly increased, from which it can be speculated that the transformation of chemical constituents during the *Citrus*-tea cofermentation procedure might be further improving the bioavailability of compounds and optimizing the flavor of pu-erh tea. Thus, more attention should be paid to the functional activities of Ganpu tea and it is of great significance to further study the correlation between biological effects and phytochemical variability of Pu-erh tea during the cofermentation process with *Citrus* peel.

## Conclusions

In this study, the non-volatile chemical profile of PE was investigated. A total of 171 compounds were identified or tentatively characterized. The chemical difference of Pu-erh tea between the raw and cofermented PE with *Citrus* peel was comprehensively compared by an integrated metabolomics strategy. The untargeted metabolomics results indicated the distinct difference between PE and Ganpu tea. The targeted comparison analysis based on the quantification of 89 main chemical constituents was quantitated. The total content of phenolic acids and flavan-3-ols dropped sharply in the Ganpu tea samples. A combination of *T*-test and multivariate statistical analysis were conducted to detect potential chemical markers and 16 chemical constituents were screened as potential chemical markers. The ability of 16 variables to correctly distinguish PE and Ganpu tea was then proved by the results of ROC analysis. Sixteen differentiated components were screened as potential markers, which showed good ability for discrimination of the two groups. The results contribute to understanding better the influence of the cofermentation processing on the chemical character of pu-erh tea.

## Data Availability Statement

The original contributions presented in the study are included in the article/Supplementary Material, further inquiries can be directed to the corresponding author/s.

## Author Contributions

YX: investigation, formal analysis, data curation, and methodology. P-LL: investigation, data curation, and validation. X-LC, M-JG, and LZ: investigation, visualization, and resources. X-HQ: writing—review and editing. JZ and Z-HH: conceptualization, investigation, and resources. WX: conceptualization, writing—original draft, project administration, funding acquisition, conceptualization, and resources. All authors contributed to the article and approved the submitted version.

## Funding

This work was supported by the special foundation of Guangzhou key laboratory (No. 202002010004), Pearl River S&T Nova Program of Guangzhou (201806010048), and Special Subject of TCM Science and Technology Research of Guangdong Provincial Hospital of Chinese Medicine (YN2018QJ07 and YN2016QJ01).

## Conflict of Interest

The authors declare that the research was conducted in the absence of any commercial or financial relationships that could be construed as a potential conflict of interest.

## Publisher's Note

All claims expressed in this article are solely those of the authors and do not necessarily represent those of their affiliated organizations, or those of the publisher, the editors and the reviewers. Any product that may be evaluated in this article, or claim that may be made by its manufacturer, is not guaranteed or endorsed by the publisher.

## Supplementary Material

Supplementary Figure 1 RSD distribution of the targets in untargeted metabolomics and quantitative analysis. **(A)** RSD distribution of 2017 features in untargeted metabolomics analysis. **(B)** RSD distribution of 89 compounds in the quantitative analysis.

Supplementary Figure 2 Two hundred iterations permutation tests of OPLS-DA model [*R*^2^ = (0.0, 0.0869), *Q*^2^ = (0.0, −0.357)].

Supplementary Figure 3 Metabolic pathway of polyphenols biosynthesis in tea plants (pen, pentose; dhex, deoxyhexose; hex, hexose; EC, epicatechin; ECG, epicatechin gallate; EGC, epigallocatechin; EGCG, epigallocatechin gallate; *p*-CoQA, *p*-coumaroylquinic acid; Components with upward arrows in red text represent compounds that significantly increased in Pu-erh tea after cofermented with *Citrus* peel).

Supplementary Table 1 Quantitative analysis results of 89 chemical components in tea samples (T = 1, *P* < 0.05; T = 0, *P* > 0.05).

Supplementary Table 2 Variable influences in projection (VIP), area under curve (AUC), and fold change (FC) values of 39 potential chemical markers (positive fold change denotes PE/Ganpu tea fold change and negative fold change denotes Ganpu tea/PE fold change).

Supplementary Table 3 Components identified or tentatively characterized in Ganpu tea (C, caffeoyl; p-Co, p-coumaroyl; Co, coumaric acid; F, feruyl; QA, quinic acid; G, galloyl; B, benzenepropanoyl; GA, gallic acid; pen, pentose; dhex, deoxyhexose; hex, hexose; GDL, D-(+)-Glucono-1, 5-lactone; glc ua, Glucuronic acid; 1 Identified by Standard substances based on level 1; 2 Identified based on level 2; 3# Potential new compounds identified in pu-erh tea based on level 3; ^*^ [M+H]+ ion).
