# Supplementary figures and images for: The Impact of Citrus-Tea Cofermentation Process on Chemical Composition and Contents of Pu-Erh Tea: An Integrated Metabolomics Study
Source: Front Nutr. 2021 Sep 17;8:737539. doi: 10.3389/fnut.2021.737539 (PMC8484324; doi:10.3389/fnut.2021.737539)

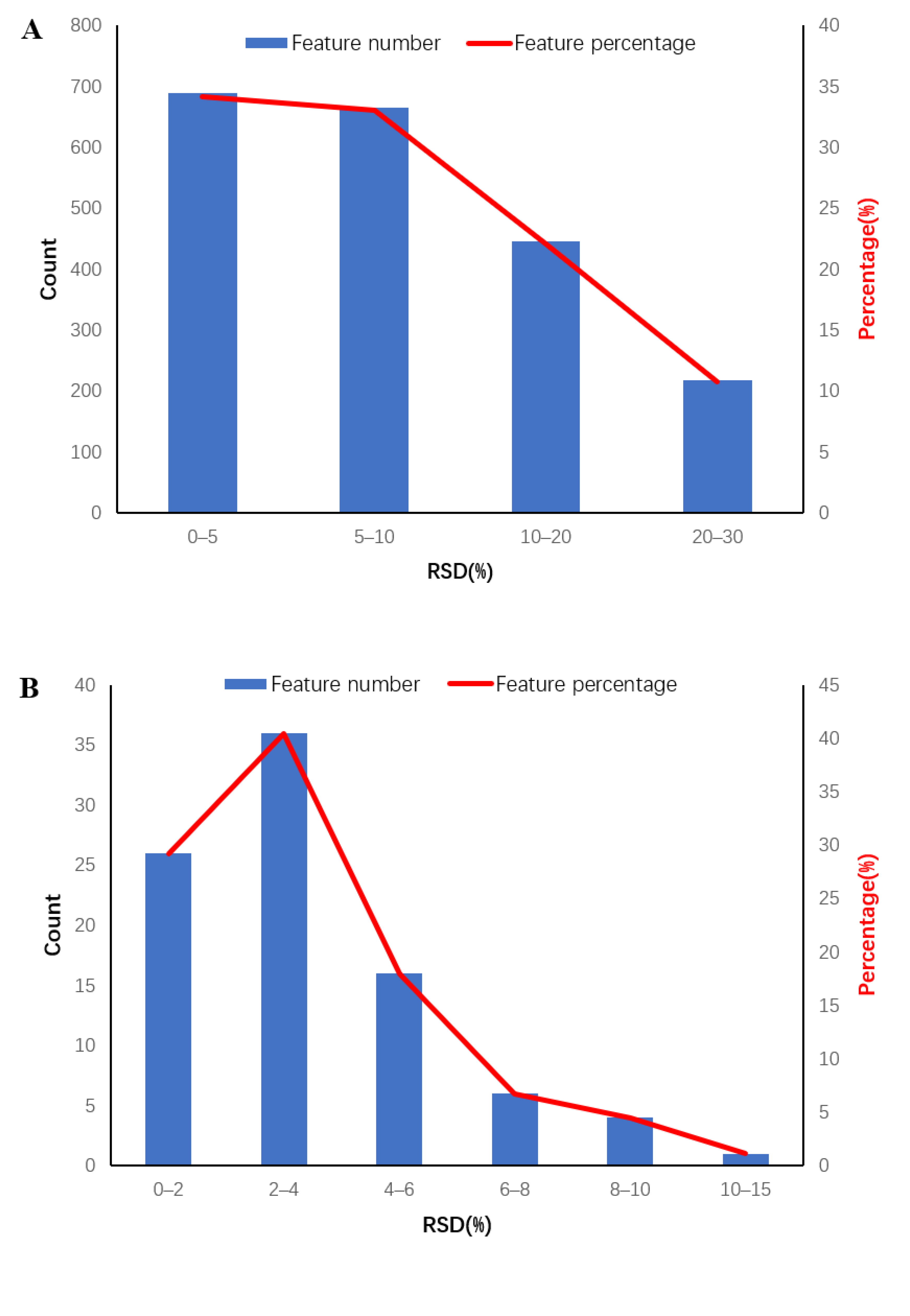

Supplement: Supplementary Figure 1 — RSD distribution of the targets in untargeted metabolomics and quantitative analysis. (A) RSD distribution of 2017 features in untargeted metabolomics analysis. (B) RSD distribution of 89 compounds in the quantitative analysis. [file Image_1.TIF]

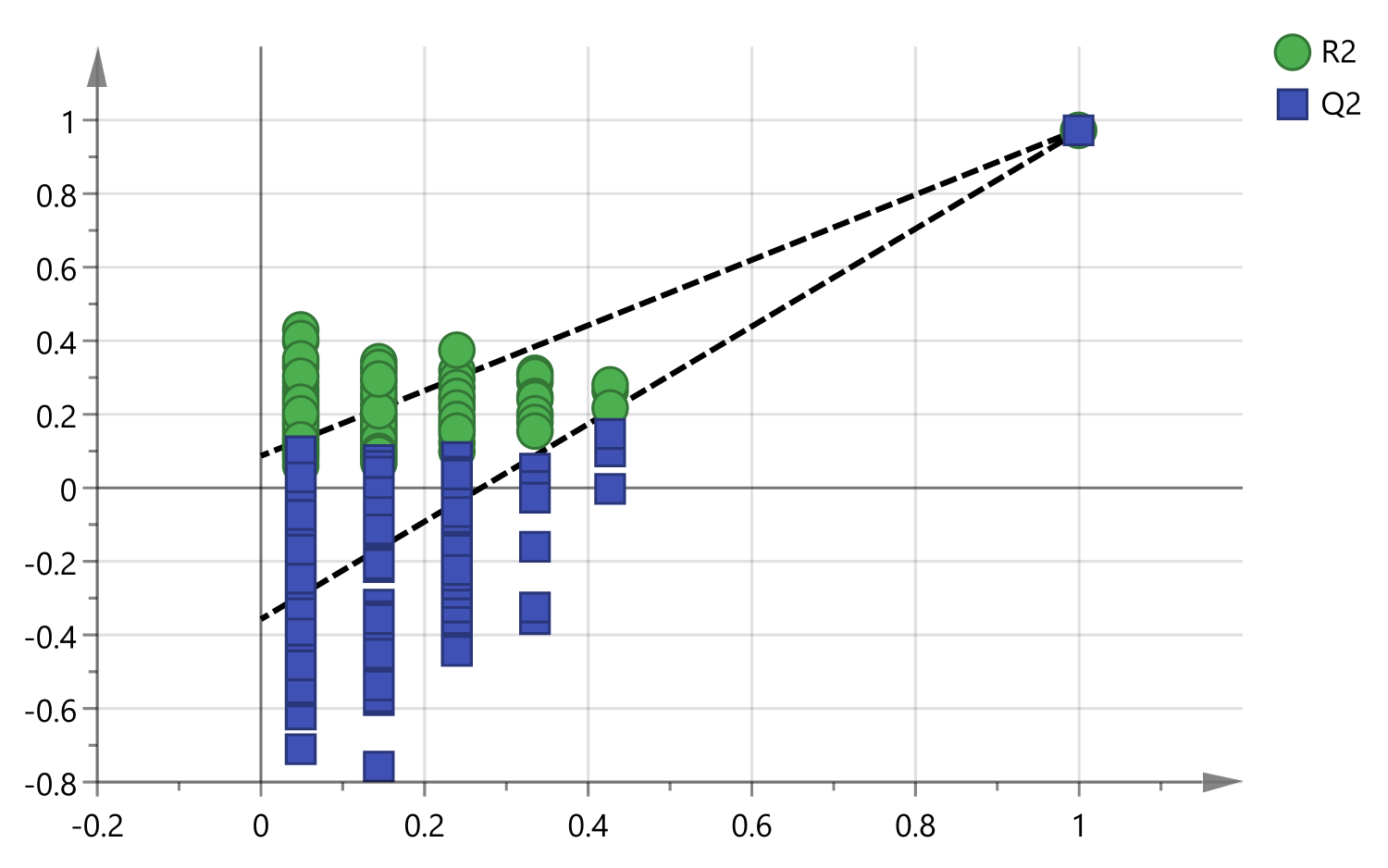

Supplement: Supplementary Figure 2 — Two hundred iterations permutation tests of OPLS-DA model [R2 = (0.0, 0.0869), Q2 = (0.0, −0.357)]. [file Image_2.TIF]

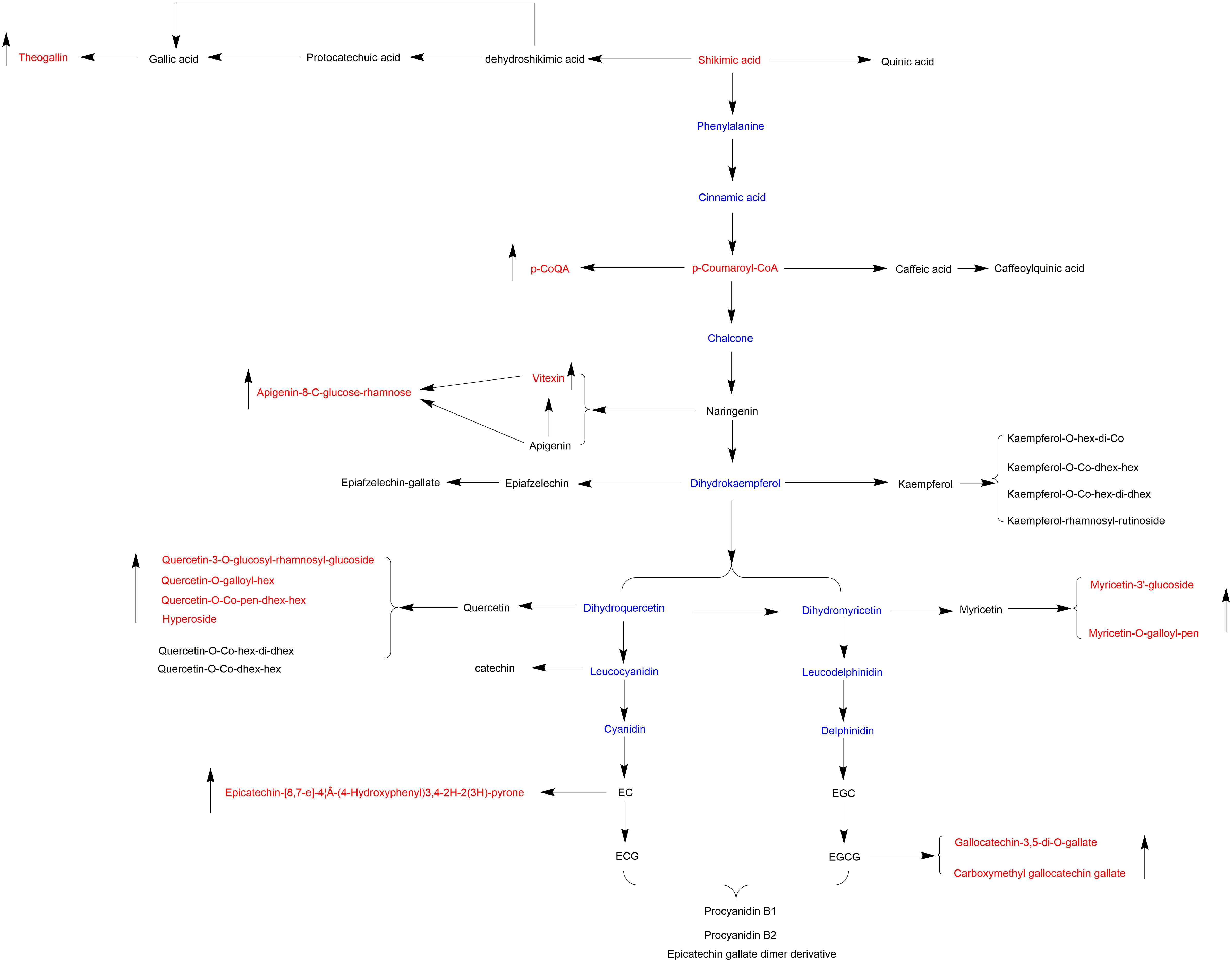

Supplement: Supplementary Figure 3 — Metabolic pathway of polyphenols biosynthesis in tea plants (pen, pentose; dhex, deoxyhexose; hex, hexose; EC, epicatechin; ECG, epicatechin gallate; EGC, epigallocatechin; EGCG, epigallocatechin gallate; p-CoQA, p-coumaroylquinic acid; Components with upward arrows in red text represent compounds that significantly increased in Pu-erh tea after cofermented with Citrus peel). [file Image_3.TIF]
