# Supplementary material for: The Impact of Citrus-Tea Cofermentation Process on Chemical Composition and Contents of Pu-Erh Tea: An Integrated Metabolomics Study
Source: Front Nutr. 2021 Sep 17;8:737539. doi: 10.3389/fnut.2021.737539 (PMC8484324; doi:10.3389/fnut.2021.737539)
Supplement: Supplementary Table 1 — Quantitative analysis results of 89 chemical components in tea samples (T = 1, P < 0.05; T = 0, P > 0.05). [file Table_1.DOCX]

**Supplementary Table 1.** Quantitative analysis results of 89 chemical components in tea samples. (T=1, P<0.05; T=0, P>0.05).

| Compounds chasses | NO | Name | Content (Mean ± SD, µg/g) | | T |
| --- | --- | --- | --- | --- | --- |
|  |  |  | PE | Ganpu tea |  |
| **Flavonoid glycosides** |  | Taxifolin-C-pen | 7.65±0.89 | 6.23±0.7 | 1 |
|  |  | Naringenin-C-di-hex | 10.08±1.19 | 6.52±0.84 | 1 |
|  |  | Isoschaftoside | 142.99±20.26 | 149.45±20.37 | 0 |
|  |  | Myricetin-O-galloyl-pen | 3.12±0.22 | 3.33±0.29 | 1 |
|  |  | Myricetin 3-robinobioside | 9.64±1.43 | 10.56±2.15 | 0 |
|  |  | Quercetin-3-O-glucosyl-rhamnosyl-glucoside | 11.93±1.79 | 15.44±3.73 | 1 |
|  |  | Myricetin-3′-glucoside | 31.48±4.25 | 37.06±5.31 | 1 |
|  |  | Quercetin-O-hex-di-dhex, | 50.64±4.51 | 51.72±8.97 | 0 |
|  |  | Apigenin-8-C-glucose-rhamnose | 7.3±1.13 | 10.39±1.46 | 1 |
|  |  | Kaempferol-rhamnosyl-rutinoside | 36.44±6.19 | 20.26±4.1 | 1 |
|  |  | Naringenin-C-hex | 11.29±1.2 | 7.93±0.6 | 1 |
|  |  | Kaempferol‐3‐O‐glucosyl‐rhamnosyl‐glucoside | 47.51±4.4 | 48.56±8.76 | 0 |
|  |  | Quercetin-O-galloyl-O-hex | 3.68±1.55 | 5.3±2.47 | 1 |
|  |  | Naringenin-O-dhex-O-hex | 3.4±3.4 | 4.43±2.63 | 1 |
|  |  | Kaempferol-7-(6″-galloylglucoside) | 3.5±1.01 | 3.57±1.15 | 0 |
|  |  | Quercetin 3-O-α-arahinoside | 13.59±2.57 | 14.78±2.96 | 0 |
|  |  | Quercetin-O-Co-hex-di-dhex | 37.36±4.93 | 31.35±4.97 | 1 |
|  |  | Quercetin-O-Co-pen-dhex-hex | 15.91±2.81 | 20.9±4.02 | 1 |
|  |  | Kaempferol-O-Co-hex-di-dhex | 14.91±2.65 | 9.07±1.85 | 1 |
|  |  | Kaempferol-C-dhex | 1.72±1.08 | 1.27±0.42 | 0 |
|  |  | Kaempferol-O-Co-pen-dhex-hex | 9.16±1.92 | 11.2±2.12 | 1 |
|  |  | Quercetin-O-Co-dhex-hex | 24.26±3.5 | 18.65±2.54 | 1 |
|  |  | Apigenin-C-pen | 62.72±8.81 | 47.28±4.99 | 1 |
|  |  | Kaempferol-O-Co-dhex-hex | 4.11±1.02 | 1.95±0.73 | 1 |
|  |  | Quercetin-O-Co-dhex-hex | 3.82±1.18 | 3.34±1.15 | 0 |
|  |  | Kaempferol-O-Co-hex | 49.25±7.32 | 42.33±6.1 | 1 |
|  |  | Kaempferol-O-hex-di-Co | 24.34±6.44 | 17.58±4.33 | 1 |
|  |  | Rutin | 358.12±35.37 | 364.36±44.13 | 0 |
|  |  | Vitexin | 55.56±4.49 | 65.84±6.65 | 1 |
|  |  | Hyperoside | 225.47±20.77 | 251.92±26.63 | 1 |
|  |  | Kaempferol-3-O-rutinoside | 143.59±9.02 | 139.54±14.63 | 0 |
|  |  | Astragalin | 143.52±14.96 | 140.64±16.04 | 0 |
|  |  | **Total content** | 1568.04±153.61 | 1562.76±187.89 |  |
| **Flavonoid aglycones** |  | Trans-3,3′,4′,5,5′,7-hexahydroxyflavanone | 6.19±0.84 | 4.44±0.35 | 1 |
|  |  | Naringenin | 7.52±0.81 | 5.3±0.48 | 1 |
|  |  | 3',4',5-Trihydroxy-7-methoxyflavone | 1.08±0.16 | 1.23±0.16 | 1 |
|  |  | 3,5,7,3’,4’-Penhydroxy-5’-methoxyflavone | 6.09±0.61 | 4.19±0.23 | 1 |
|  |  | Taxifolin | 3.24±0.46 | 1.63±0.27 | 1 |
|  |  | Myricetin | 46.08±4.45 | 33.12±4.67 | 1 |
|  |  | Luteolin | 6.55±0.79 | 6.96±0.82 | 0 |
|  |  | Quercetin | 254.27±33.55 | 220.65±31.39 | 1 |
|  |  | Apigenin | 1.88±0.34 | 1.81±0.26 | 0 |
|  |  | Kaempferol | 83.57±14.04 | 69.29±10.99 | 1 |
|  |  | **Total content** | 416.47±54.86 | 348.62±48.19 |  |
| **Phenolic acids** |  | Quinic acid-O-dhex | 1.84±0.51 | 2±0.54 | 0 |
|  |  | Theogallin | 78.02±13.38 | 113.91±26.98 | 1 |
|  |  | Dihydroxyphenyl propionic acid | 4±0.53 | 2.07±0.22 | 1 |
|  |  | p-CoQA-1 | 88.41±11 | 111.08±12.53 | 1 |
|  |  | Salicylic acid | 31.24±1.76 | 46.49±2.88 | 1 |
|  |  | p-CoQA-2 | 171.95±18.4 | 232.96±22.22 | 1 |
|  |  | 3-B-4-Caffeoylquinic acid | 131.85±17.52 | 131.22±16.32 | 0 |
|  |  | Caffeic acid-C-hex | 15.8±1.65 | 11.28±0.66 | 1 |
|  |  | 1-C-3-p-CoQA | 3.51±0.47 | 3.08±0.55 | 1 |
|  |  | 1,3/3,5-di-p-CoQA | 2.61±0.91 | 2.64±0.83 | 0 |
|  |  | 4-Hydroxy-3,5-dimethoxycinnamic acid | 0.84±0.16 | 2.04±0.26 | 1 |
|  |  | Quinic acid | 790.85±52.73 | 690.56±101.51 | 1 |
|  |  | Gallic acid | 3533.17±317.81 | 1635.05±211.91 | 1 |
|  |  | Neochlorogenic acid | 46.82±6.44 | 31.59±5.14 | 1 |
|  |  | Protocatechuic acid | 155.67±13.24 | 117.51±9.86 | 1 |
|  |  | Chlorogenic acid | 182.73±31.63 | 133.69±25.09 | 1 |
|  |  | Caffeic acid | 2.64±0.52 | 2.11±0.25 | 1 |
|  |  | trans-p-Coumaric acid | 10.49±1.05 | 19.15±1.28 | 1 |
|  |  | Shikimic acid | 11.49±1.26 | 14.44±1.37 | 1 |
|  |  | **Total content** | 5263.93±417.64 | 3302.87±388.03 |  |
| **Flavan-3-ols** |  | (-)-Gallocatechin-glc ua | 16.94±4.02 | 8.62±1.08 | 1 |
|  |  | Catechin-C-hex | 20±4.89 | 5.05±3.28 | 1 |
|  |  | Catechin-glc ua | 25.9±8.47 | 9.05±4.78 | 1 |
|  |  | Epicatechin-(4beta- > 8)-epigallocatechin 3-O-gallate | 16.05±3.34 | 14.09±3.65 | 0 |
|  |  | 6-Carboxyl-(-)-Gallocatechin | 167.45±14.42 | 123.34±9.82 | 1 |
|  |  | Ent-epicatechin-(4alpha- > 8)-ent-epicatechin 3-gallate | 15.78±3.66 | 14.41±5.09 | 0 |
|  |  | 8-carboxyl-(+)-catechin | 501.1±51.19 | 242.91±25.53 | 1 |
|  |  | Carboxymethyl gallocatechin gallate | 4.79±0.4 | 6.86±0.8 | 1 |
|  |  | Gallocatechin-3,5-di-O-gallate | 4.41±0.97 | 5.96±1.43 | 1 |
|  |  | Epicatechin gallate dimer derivative | 31.42±39.61 | 12.27±10.81 | 1 |
|  |  | Epicatechin-[8,7-e]-4β-(4-Hydroxyphenyl)3,4-2H-2(3H)-pyrone-1 | 26.02±4.24 | 41.08±6.59 | 1 |
|  |  | Epicatechin-[8,7-e]-4β-(4-Hydroxyphenyl)3,4-2H-2(3H)-pyrone-2 | 54.04±7.82 | 76.37±12.2 | 1 |
|  |  | (Epi)gallocatechin–(epi)catechin isomer | 52.73±9.08 | 24.58±5.48 | 1 |
|  |  | Chalcan-flavan dimers | 34.83±11.64 | 6.03±1.17 | 1 |
|  |  | Gallocatechin-CH_2_-gallocatechin gallate | 22.81±8 | 10±2.06 | 1 |
|  |  | (-)-Gallocatechin | 224.41±34.25 | 72.98±15.14 | 1 |
|  |  | Procyanidin B1 | 17.36±2.61 | 10.45±1.94 | 1 |
|  |  | Epigallocatechin | 254.16±45.65 | 61.23±16.49 | 1 |
|  |  | Procyanidin B2 | 168.38±27.75 | 79.27±16.18 | 1 |
|  |  | Epicatechin | 666.22±86.95 | 193.6±32.06 | 1 |
|  |  | Epigallocatechin gallate | 169.98±44.86 | 122.06±23.18 | 1 |
|  |  | (-)-Gallocatechin 3-O-gallate | 49.73±11.57 | 50.74±8.59 | 0 |
|  |  | Epicatechin gallate | 308.82±147.85 | 229.49±61.66 | 1 |
|  |  | Epiafzelechin | 12.61±1.51 | 5.86±0.38 | 1 |
|  |  | Epiafzelechin-gallate | 19.84±4.72 | 15.08±3.08 | 1 |
|  |  | **Total content** | 2885.77±350.81 | 1441.38±216.61 |  |
| **Others** |  | L-Theanine | 16.84±3.49 | 7.09±1.14 | 1 |
|  |  | Theobromine | 1768.65±237.06 | 1889.17±259.09 | 0 |
|  |  | Caffeine | 38403.86±5102.55 | 38694.2±6647.21 | 0 |
