# Supplementary material for: The Impact of Citrus-Tea Cofermentation Process on Chemical Composition and Contents of Pu-Erh Tea: An Integrated Metabolomics Study
Source: Front Nutr. 2021 Sep 17;8:737539. doi: 10.3389/fnut.2021.737539 (PMC8484324; doi:10.3389/fnut.2021.737539)
Supplement: Supplementary Table 2 — Variable influences in projection (VIP), area under curve (AUC), and fold change (FC) values of 39 potential chemical markers (positive fold change denotes PE/Ganpu tea fold change and negative fold change denotes Ganpu tea/PE fold change). [file Table_2.DOCX]

**Supplementary Table 2.** Variable influences in projection (VIP), Area under curve (AUC) and Fold change (FC) values of 39 potential chemical markers. (Positive fold change denotes PE/Ganpu tea fold change, Negative fold change denotes Ganpu tea/PE fold change)

| No | Name | VIP | AUC | FC |
| --- | --- | --- | --- | --- |
|  | Epicatechin | 1.188 | 1.000 | 3.441 |
|  | Epigallocatechin | 1.186 | 1.000 | 4.151 |
|  | (-)-Gallocatechin | 1.186 | 1.000 | 3.075 |
|  | 8-carboxyl-(+)-catechin | 1.184 | 1.000 | 2.063 |
|  | Gallic acid | 1.169 | 0.812 | 1.145 |
|  | Epiafzelechin | 1.165 | 1.000 | 2.152 |
|  | 3,5,7,3’,4’-Penhydroxy-5’-methoxyflavone | 1.156 | 1.000 | 1.455 |
|  | Dihydroxyphenyl propionic acid | 1.154 | 1.000 | 1.935 |
|  | Taxifolin | 1.148 | 1.000 | 1.991 |
|  | (Epi)gallocatechin–(epi)catechin isomer | 1.145 | 0.995 | 2.145 |
|  | Procyanidin B2 | 1.143 | 0.998 | 2.124 |
|  | Chalcan-flavan dimers | 1.131 | 1.000 | 5.778 |
|  | Naringenin-C-hex | 1.130 | 0.991 | 1.423 |
|  | Naringenin-C-di-hex | 1.128 | 1.000 | 1.545 |
|  | Catechin-C-hex | 1.125 | 1.000 | 3.960 |
|  | Caffeic acid-C-hex | 1.121 | 0.998 | 1.400 |
|  | L-Theanine | 1.116 | 1.000 | 2.375 |
|  | Naringenin | 1.115 | 0.993 | 1.418 |
|  | 6-Carboxyl-(-)-Gallocatechin | 1.105 | 0.991 | 1.358 |
|  | (-)-Gallocatechin-glc ua | 1.098 | 1.000 | 1.965 |
|  | Protocatechuic acid | 1.095 | 1.000 | 1.325 |
|  | Procyanidin B1 | 1.090 | 0.973 | 1.661 |
|  | Trans-3,3′,4′,5,5′,7-hexahydroxyflavanone | 1.086 | 0.986 | 1.395 |
|  | Neochlorogenic acid | 1.076 | 0.973 | 1.482 |
|  | Catechin-glc ua | 1.071 | 0.966 | 2.861 |
|  | Myricetin | 1.068 | 0.957 | 1.391 |
|  | Kaempferol-O-Co-hex-di-dhex | 1.067 | 0.966 | 1.645 |
|  | Kaempferol-rhamnosyl-rutinoside | 1.020 | 1.000 | 1.798 |
|  | Quercetin-O-Co-dhex-hex | 1.003 | 0.893 | 1.301 |
|  | KaempferolO-Co-dhex-hex | 1.064 | 0.771 | –1.224 |
|  | trans-p-Coumaric acid | 1.175 | 1.000 | –1.826 |
|  | 4-Hydroxy-3,5-dimethoxycinnamic acid | 1.172 | 1.000 | –2.436 |
|  | Salicylic acid | 1.158 | 1.000 | –1.488 |
|  | p-CoQA-2 | 1.095 | 0.989 | –1.355 |
|  | Carboxymethyl gallocatechin gallate | 1.085 | 1.000 | –1.432 |
|  | Epicatechin-[8,7-e]-4β-(4-Hydroxyphenyl)3,4-2H-2(3H)-pyrone-1 | 1.061 | 0.989 | –1.579 |
|  | Epicatechin-[8,7-e]-4β-(4-Hydroxyphenyl)3,4-2H-2(3H)-pyrone-2 | 1.027 | 0.973 | –1.413 |
|  | Shikimic acid | 1.024 | 0.966 | –1.257 |
|  | Apigenin-8-C-glucose-rhamnose | 1.014 | 0.952 | –1.423 |
