# Supplementary material for: The Impact of Citrus-Tea Cofermentation Process on Chemical Composition and Contents of Pu-Erh Tea: An Integrated Metabolomics Study
Source: Front Nutr. 2021 Sep 17;8:737539. doi: 10.3389/fnut.2021.737539 (PMC8484324; doi:10.3389/fnut.2021.737539)
Supplement: Supplementary Table 3 — Components identified or tentatively characterized in Ganpu tea (C, caffeoyl; p-Co, p-coumaroyl; Co, coumaric acid; F, feruyl; QA, quinic acid; G, galloyl; B, benzenepropanoyl; GA, gallic acid; pen, pentose; dhex, deoxyhexose; hex, hexose; GDL, D-(+)-Glucono-1, 5-lactone; glc ua, Glucuronic acid; 1 Identified by Standard substances based on level 1; 2 Identified based on level 2; 3# Potential new compounds identified in pu-erh tea based on level 3; * [M+H]+ ion). [file Table_3.DOCX]

**Supplementary Table 3.** Components identified or tentatively characterized in Ganpu tea.

| NO | RT | [M–H]^–^ | Molecular formula | MS/MS | Identiﬁcation |
| --- | --- | --- | --- | --- | --- |
|  | 1.29 | 353.1086 | C_13_H_21_O_11_ | 191.0552, 179.0550, 147.0652 | ^3#^Quinic acid-O-hex |
|  | 1.34 | 339.0741 | C_15_H_15_O_9_ | 177.0181 | ^2^Esculin |
|  | 1.35 | 191.0552 | C_7_H_11_O_6_ | 173.0446, 147.0646 | ^1^Quinic acid |
|  | 1.37 | 435.0915 | C_20_H_19_O_11_ | 303.0519, 261.0412, 217.0524, 125.0228 | ^3#^Taxifolin-C-pen |
|  | 1.40 | 175.1079* | C_7_H_15_O_3_N_2_ | 158.0815, 128.0709, 84.0451 | ^1^L-Theanine |
|  | 1.41 | 337.1139 | C_13_H_21_O_10_ | 247.0823, 191.0551, 173.0444, 147.0650 | ^2^Quinic acid-O-dhex |
|  | 1.41 | 477.1255 | C_19_H_25_O_14_ | 169.0132, 125.023 | ^3#^Gallic acid-O-rutinoside |
|  | 1.75 | 133.0131 | C_4_H_5_O_5_ | 107.0349, 92.0239 | ^2^Malic acid |
|  | 1.89 | 191.0189 | C_6_H_7_O_7_ | 173.0080, 129.0179, 111.0073 | ^2^Citric acid |
|  | 1.91 | 331.0667 | C_13_H_15_O_10_ | 169.0132, 151.0028, 125.0230 | ^2^6-O-Galloylglucose |
|  | 1.93 | 477.1255 | C_19_H_25_O_14_ | 169.0132, 125.0232 | ^3#^Gallic acid-O-rutinoside |
|  | 2.21 | 343.0667 | C_14_H_15_O_10_ | 191.0552, 173.0449, 169.0131, 125.0231 | ^2^Theogallin |
|  | 2.5 | 169.0132 | C_7_H_5_O_5_ | 125.0230 | ^1^Gallic acid |
|  | 3.0 | 181.0719* | C_7_H_9_O_2_N_4_ | 163.0615, 137.0822, 96.0567 | ^1^Theobromine |
|  | 3.15 | 481.0993 | C_21_H_21_O_13_ | 377.0868, 347.0770, 329.0666, 305.0664, 285.0768, 261.0765, 221.0449, 125.0230 | ^3#^(-)-Gallocatechin-glc ua |
|  | 3.42 | 261.0768 | C_14_H_13_O_5_ | 243.0657, 219.0655, 177.0182 | ^2^Celereoin |
|  | 3.53 | 305.0669 | C_15_H_13_O_7_ | 219.0656, 179.0341, 167.0340, 137.0231, 125.0231 | ^1^(-)-Gallocatechin |
|  | 4.03 | 165.0547 | C_9_H_9_O_3_ | 147.0440, 121.0279 | ^2^3-(4-Hydroxyphenyl) propionic acid |
|  | 4.13 | 223.0608 | C_11_H_11_O_5_ | 195.0655, 179.0701, 161.0597 | ^3#^4-Hydroxy-3, 5-dimethoxycinnamic acid |
|  | 4.2 | 353.0877 | C_16_H_17_O_9_ | 191.0553, 179.0341, 173.0445, 135.0438 | ^1^Neochlorogenic acid |
|  | 4.23 | 153.0182 | C_7_H_5_O_4_ | 109.0281 | ^1^Protocatechuic acid |
|  | 4.29 | 315.0751 | C_13_H_15_O_9_ | 271.0416, 241.0021, 153.0181, 109.0277 | ^3#^Protocatechuic acid-hex |
|  | 4.56 | 641.1364 | C_27_H_29_O_18_ | 403.1029, 373.0931, 331.0816, 289.0713, 205.0500, 125.0230 | ^3#^(Epi)catechin-di-glc ua |
|  | 4.62 | 577.1356 | C_30_H_25_O_12_ | 407.0770, 289.0720, 245.0820, 205.0502, 125.0232 | ^1^Procyanidin B1 |
|  | 4.66 | 181.0497 | C_9_H_9_O_4_ | 163.0390, 135.0439, 119.0489 | ^2^4-Hydroxy-3-methoxybenzeneacetic acid |
|  | 4.67 | 451.1249 | C_21_H_23_O_11_ | 361.0918, 331.0821, 289.0714, 245.0813, 205.0497, 125.0233 | ^2^Catechin-C-hex |
|  | 4.68 | 331.0819 | C_17_H_15_O_7_ | 289.0719, 245.0817, 205.0498, 125.0231 | ^3#^Acetylated (epi)catechin |
|  | 4.74 | 483.0782 | C_20_H_19_O_14_ | 331.0674, 271.0464, 211.0240, 169.0133, 125.0233 | ^2^di-Galloylglucose isomer |
|  | 4.74 | 183.0291 | C_8_H_7_O_5_ | 168.0054, 139.0389, 124.0153 | ^2^4-O-Methylgallic acid |
|  | 4.75 | 305.0668 | C_15_H_13_O_7_ | 287.0533, 261.0768, 219.0656, 179.0341, 125.0231 | ^1^(-)-Epigallocatechin |
|  | 4.77 | 261.0768 | C_14_H_13_O_5_ | 243.0657, 219.0655, 177.0182 | ^2^Celereoin isomer |
|  | 4.77 | 593.1299 | C_30_H_25_O_13_ | 305.0667, 289.0723, 245.0814, 219.0655, 177.0183, 125.0231 | ^2^ (Epi)Gallocatechin–(epi)catechin isomer |
|  | 4.77 | 495.0787 | C_21_H_19_O_14_ | 343.0668, 191.0556, 169.0133, 125.0230 | ^2^di-GQA |
|  | 4.77 | 611.1401 | C_30_H_27_O_14_ | 305.0672, 137.0229, 125.0234 | ^2^Chalcan-flavan dimers |
|  | 4.82 | 641.1364 | C_27_H_29_O_18_ | 403.1029, 373.0931, 331.0816, 289.0713, 205.0500, 125.0229 | ^3#^(Epi)catechin-di-glc ua |
|  | 4.83 | 319.0458 | C_15_H_11_O_8_ | 233.1047, 217.0134, 191.0340, 177.0182, 153.0183 | ^2^Trans-3, 3′, 4′, 5, 5′, 7-hexahydroxyﬂavanone |
|  | 4.83 | 275.0559 | C_14_H_11_O_6_ | 247.0247, 231.0656, 163.0754, 125.0230 | ^2^Teadenol A/B |
|  | 4.95 | 465.1040 | C_21_H_21_O_12_ | 361.0930, 331.0823, 301.0716, 289.0719, 245.0813, 205.0496, 179.0338, 125.0232 | ^3#^Catechin-glc ua |
|  | 5.01 | 337.0927 | C_16_H_17_O_8_ | 191.0552, 173.0445, 163.0389, 119.049 | ^2^p-CoQA-1 |
|  | 5.04 | 633.0733 | C_27_H_21_O_18_ | 463.0521, 347.0780, 300.9989, 169.0134, 125.0230 | ^2^3-O-galloyl-4, 6(S)-hexahydroxydiphenoyl-D-glucose |
|  | 5.07 | 353.0877 | C_16_H_17_O_9_ | 191.0553, 179.0341, 173.0445, 135.0438 | ^1^Chlorogenic acid |
|  | 5.10 | 595.1672 | C_27_H_31_O_15_ | 415.1034, 385.0924, 355.0817, 313.0713, 271.0612, 125.0232 | ^3#^Naringenin-C-di-hex |
|  | 5.36 | 183.0290 | C_8_H_7_O_5_ | 168.0054, 139.0389, 124.0153 | ^2^4-O-Methylgallic acid isomer |
|  | 5.15 | 369.0283 | C_15_H_13_O_9_S | 289.0714, 245.0813, 205.0497, 125.0233 | ^2^Catechin sulfide |
|  | 5.17 | 449.1082 | C_21_H_21_O_11_ | 359.0774, 289.0727, 205.0504, 125.0229 | ^3#^Catechin-GDL |
|  | 5.17 | 353.0877 | C_16_H_17_O_9_ | 191.0553, 179.0341, 173.0445, 135.0438 | ^1^Cryptochlorogenic acid |
|  | 5.18 | 515.1418 | C_22_H_27_O_14_ | 191.0553, 163.0389 | ^2^Caffeoylquinic acid-O-hex |
|  | 5.19 | 195.0876* | C_8_H_11_O_2_N_4_ | 138.0662, 110.0714 | ^1^Caffeine |
|  | 5.19 | 289.0718 | C_15_H_13_O_6_ | 245.0818, 203.0703, 179.0341, 151.0387, 125.0231, 109.0281 | ^2^Catechin |
|  | 5.21 | 367.1030 | C_17_H_19_O_9_ | 193.0497, 191.0551, 173.0444, 149.0595 | ^2^Feruloylquinic acid |
|  | 5.23 | 385.0232 | C_15_H_13_O_10_S | 305.0666, 217.0132, 137.0231, 125.0231 | ^2^Gallocatechin sulfide |
|  | 5.29 | 547.1034 | C_21_H_19_O_9_ | 253.0937 | ^1^Puerarin |
|  | 5.30 | 137.0233 | C_7_H_5_O_3_ | 119.0124, 109.0280, 93.0331 | ^2^Salicylic acid |
|  | 5.32 | 577.1356 | C_30_H_25_O_12_ | 407.0770, 289.0720, 245.0820, 205.0502, 125.0232 | ^1^Procyanidin B2 |
|  | 5.42 | 745.1414 | C_37_H_29_O_17_ | 423.0725, 407.0774, 305.0665, 289.0721, 177.0184, 169.0132, 125.0231 | ^2^Epicatechin-(4beta- > 8)-epigallocatechin 3-O-gallate |
|  | 5.43 | 349.0585 | C_16_H_13_O_9_ | 331.0455, 305.0674, 287.0551, 245.0452, 205.0129, 125.0231 | ^2^6-Carboxyl-(-)-Gallocatechin |
|  | 5.44 | 623.1625 | C_28_H_31_O_16_ | 503.1201, 463.0673, 413.0879, 383.0772, 341.0649, 315.0506, 151.0024 | ^3#^Isorhamnetin-C-hex-dhex |
|  | 5.52 | 369.0283 | C_15_H_13_O_9_S | 289.0714, 245.0813, 205.0497, 125.0233 | ^2^Epicatechin sulfide |
|  | 5.53 | 467.1195 | C_21_H_23_O_12_ | 377.0866, 347.0768, 305.0662, 221.0447, 167.0336, 125.0231 | (-)-Gallocatechin-8-C-β-D-glucopyranoside |
|  | 5.59 | 563.1406 | C_26_H_27_O_14_ | 383.0769, 353.0664, 293.0456, 269.0460, 125.0234 | ^2^Isoschaftoside |
|  | 5.60 | 635.0894 | C_27_H_23_O_18_ | 465.0682, 313.0563, 169.0132, 125.0231 | ^2^1, 4, 6-tri-O-galloyl-beta-D-glucopyranose |
|  | 5.62 | 289.0718 | C_15_H_13_O_6_ | 245.0818, 203.0703, 179.0341, 151.0387, 125.0231, 109.0281 | ^1^Epicatechin |
|  | 5.63 | 385.0232 | C_15_H_13_O_10_S | 305.0666, 217.0132, 137.0231, 125.0231 | ^2^Epigallocatechin sulfide |
|  | 5.66 | 631.0941 | C_28_H_23_O_17_ | 479.0831, 317.0287, 316.0223, 169.0131, 125.0232 | ^3#^Myricetin-O-galloyl-pen |
|  | 5.67 | 173.0444 | C_7_H_9_O_5_ | 155.0338, 137.0232, 93.0330, 59.0123 | ^2^Shikimic acid |
|  | 5.69 | 337.0927 | C_16_H_17_O_8_ | 191.0552, 173.0445, 163.0389, 119.0488 | ^2^p-CoQA-2 |
|  | 5.69 | 625.1408 | C_27_H_29_O_17_ | 316.0220, 300.0263, 271.0251, 125.0227 | ^2^Myricetin 3-robinobioside |
|  | 5.70 | 179.0340 | C_9_H_7_O_4_ | 164.0106, 135.0439 | ^1^Caffeic acid |
|  | 5.71 | 457.0779 | C_22_H_17_O_11_ | 305.0657, 287.0564, 219.0659, 193.0131, 169.0131, 125.0230 | ^1^Epigallocatechin gallate |
|  | 5.72 | 347.0769 | C_17_H_15_O_8_ | 329.0662, 289.0719, 245.0820, 151.0025 | ^2^6/8-Carboxymethyl-(+)-Catechin |
|  | 5.78 | 617.1143 | C_28_H_25_O_16_ | 361.0928, 331.0821, 301.0693, 289.0722, 245.0824, 169.0132, 125.0232 | ^3#^Catechin gallate-glc ua |
|  | 5.80 | 771.1995 | C_33_H_39_O_21_ | 300.0272, 301.0345, 271.0242, 151.0026 | ^2^Quercetin-3-O-glucosyl-rhamnosyl-glucoside |
|  | 5.82 | 457.0781 | C_22_H_17_O_11_ | 305.0657, 287.0564, 219.0659, 193.0131, 169.0131, 125.0230 | ^1^(-)-Gallocatechin gallate |
|  | 5.84 | 729.1466 | C_37_H_29_O_16_ | 407.0769, 339.0877, 289.0714, 245.0814, 169.0130, 125.0229 | ^2^Ent-epicatechin-(4alpha-> 8)-ent-epicatechin 3-gallate |
|  | 5.85 | 367.1030 | C_17_H_19_O_9_ | 193.0497, 191.0551, 173.0444, 149.0595 | ^2^Feruloylquinic acid |
|  | 5.86 | 479.0833 | C_21_H_19_O_13_ | 316.0219, 317.0273, 271.0246, 151.0021, 125.0220 | ^2^Myricetin-3′-glucoside |
|  | 5.87 | 755.2045 | C_33_H_39_O_20_ | 609.1455, 301.0344, 300.0272, 271.0255, 151.0019 | ^2^Quercetin-O-hex-di-dhex |
|  | 5.90 | 361.0922 | C_18_H_17_O_8_ | 343.0814, 317.0662, 289.0718, 247.0612, 205.0499 | ^2^8-Carboxymethyl-(+)-Catechin Methyl Ester |
|  | 5.90 | 533.1304 | C_25_H_25_O_13_ | 473.1095, 443.0979, 383.0770, 353.0661, 325.0712, 191.0335 | ^2^3-B-4-Caffeoylquinic acid |
|  | 5.92 | 577.1578 | C_27_H_29_O_14_ | 413.0878, 383.0762, 353.0653, 293.0457, 269.0463, 161.0239, 125.0230 | ^2^Apigenin-8-C-glucose-rhamnose |
|  | 5.93 | 739.2098 | C_33_H_39_O_19_ | 285.0404, 255.0304, 177.0183, 125.0231 | ^2^Kaempferol-rhamnosyl-rutinoside |
|  | 5.99 | 609.1464 | C_27_H_29_O_16_ | 301.0344, 300.0276, 271.0252, 151.0025 | ^1^Rutin |
|  | 6.01 | 433.1141 | C_21_H_21_O_10_ | 343.0818, 313.0717, 283.0602, 271.0611, 193.0133, 119.0488 | ^2^Naringenin-C-hex |
|  | 6.02 | 755.2045 | C_33_H_39_O_20_ | 285.0405, 255.0309, 255.0309, 229.0496, 125.0232 | ^2^Kaempferol‐3‐O‐glucosyl‐rhamnosyl‐glucoside |
|  | 6.03 | 597.1837 | C_27_H_33_O_15_ | 417.1187, 387.1085, 357.0978, 315.0877, 289.0724, 209.0447, 125.0232 | ^3#^Catechin-C-dhex-hex |
|  | 6.06 | 615.0993 | C_28_H_23_O_16_ | 463.0886, 343.0453, 301.0346, 300.0273, 283.0245, 169.0131, 151.0023, 125.0229 | ^2^Quercetin-O-galloyl-hex |
|  | 6.12 | 431.0983 | C_21_H_19_O_10_ | 341.0664, 311.0560, 283.0614, 269.0459, 109.0281 | ^1^Vitexin |
|  | 6.13 | 449.0713 | C_20_H_17_O_12_ | 317.0267, 316.0220, 271.0244, 151.0026, 125.0230 | ^3#^Myricetin-O-pen |
|  | 6.14 | 273.0767 | C_15_H_13_O_5_ | 255.0661, 205.0862, 139.0388, 97.0280 | ^2^Epiafzelechin |
|  | 6.14 | 341.0907 | C_15_H_17_O_9_ | 221.0487, 191.0339, 179.0348, 101.0230 | ^3#^Caffeic acid-C-hex |
|  | 6.14 | 333.0613 | C_16_H_13_O_8_ | 315.0511, 289.0705, 271.0613, 229.0501, 165.0183, 125.0233 | ^2^8-carboxyl-(+)-catechin |
|  | 6.14 | 881.1585 | C_44_H_33_O_20_ | 407.0774, 289.0719, 269.0458, 193.0139, 169.0134, 125.0231 | ^2^Epicatechin gallate dimer |
|  | 6.20 | 725.1942 | C_32_H_37_O_19_ | 285.0405, 257.0457, 229.0502, 125.0231 | ^3#^Kaempferol-O-pen-dhex-hex |
|  | 6.24 | 463.0886 | C_21_H_19_O_12_ | 301.0342, 300.0274, 271.0250, 255.0300, 151.0024, 125.0229 | ^1^Hyperoside/Isoquercitrin |
|  | 6.27 | 761.1564 | C_34_H_33_O_20_ | 609.1462, 357.0824, 301.0348, 300.0273, 169.0131, 125.0228 | ^3#^Rutin-O-galloyl |
|  | 6.28 | 593.1517 | C_27_H_29_HO_15_ | 285.0403, 255.0303, 229.0507, 151.0028 | ^1^Kaempferol-3-O-rutinoside |
|  | 6.30 | 499.0882 | C_24_H_19_O_12_ | 347.0768, 329.0663, 303.0875, 285.0771, 259.0975, 169.0133, 125.0231, 109.0279 | ^3#^Carboxymethyl gallocatechin gallate |
|  | 6.31 | 489.1042 | C_23_H_21_O_12_ | 191.0552, 179.0342, 163.0391, 151.0392 | ^2^Gallic acid-p-CoQA |
|  | 6.32 | 623.1625 | C_28_H_31_O_16_ | 503.1201, 463.0673, 413.0879, 383.0772, 341.0649, 315.0506, 151.0024 | ^3#^Isorhamnetin-C-hex-dhex |
|  | 6.33 | 301.9990 | C_14_H_5_O_8_ | 283.0240, 257.0079, 229.0138 | ^2^Ellagic acid |
|  | 6.33 | 467.0982 | C_24_H_19_O_10_ | 341.0664, 289.0719, 271.0613, 217.0138, 179.0338, 125.0231, 109.0280 | ^2^ (-)-Epigallocatechin-3-O-caﬀeoate |
|  | 6.36 | 609.0875 | C_29_H_21_O_15_ | 315.0511, 305.0664, 300.0270, 287.0568, 169.0132, 125.0230 | ^2^Gallocatechin-3, 5-di-O-gallate |
|  | 6.37 | 775.1520 | C_38_H_31_O_18_ | 333.0614, 315.0512, 305.0695, 289.0724, 229.0489, 169.0130, 125.0229 | ^3#^Gallocatechin-CH_2_-gallocatechin gallate |
|  | 6.38 | 579.1730 | C_27_H_31_O_14_ | 271.0617, 227.0707, 175.0027, 151.0026, 119.0489 | ^3#^Naringenin-O-dhex-hex |
|  | 6.38 | 883.1730 | C_44_H_35_O_20_ | 289.0719, 245.0825, 193.0134, 169.0131, 125.0231 | ^2^Epicatechin gallate dimer derivative |
|  | 6.38 | 441.0829 | C_22_H_17_O_10_ | 289.0719, 245.0820, 193.0134, 169.0132, 125.0231 | ^1^Epicatechin gallate |
|  | 6.4 | 331.0458 | C_16_H_11_O_8_ | 315.0512, 300.0272, 271.0252, 179.0333, 181.0134, 151.0025 | ^3#^3, 5, 7, 3’, 4’-Penhydroxy-5’-methoxyflavone |
|  | 6.44 | 561.1256 | C_26_H_25_O_14_ | 193.0497, 175.0391, 147.0284, 129.0180 | ^3#^Di-ferulic acid-D-Glucaric acid |
|  | 6.45 | 599.1044 | C_28_H_23_O_15_ | 447.0933, 313.0563, 285.0401, 241.0348, 169.0132, 125.0230 | ^2^Kaempferol-7-(6″-galloylglucoside) |
|  | 6.47 | 451.1036 | C_24_H_19_O_9_ | 341.0664, 311.0557, 305.0665, 217.0135, 177.0183, 163.0390, 145.0282, 109.0282 | ^2^ (Epi)gallocatechin 3-O-p-coumaroate isomer |
|  | 6.48 | 163.0391 | C_9_H_7_O_3_ | 147.0440, 135.0074, 119.0489, | ^1^trans-p-Coumaric acid |
|  | 6.50 | 433.0776 | C_20_H_17_O_11_ | 343.0455, 300.0274, 301.0338, 271.0252, 151.0026 | ^2^Quercetin 3-O-α-arahinoside |
|  | 6.55 | 447.0937 | C_21_H_19_O_11_ | 285.0392, 284.0328, 255.0298, 227.0341, 151.0024 | ^1^Astragaline |
|  | 6.56 | 609.1827 | C_28_H_33_O_15_ | 301.0713, 242.0582, 151.0024, 125.0229 | ^3#^Hesperidin |
|  | 6.59 | 183.0291 | C_8_H_7_O_5_ | 168.0054, 139.0389, 124.0153 | ^2^4-O-Methylgallic acid isomer |
|  | 6.67 | 505.0993 | C_23_H_21_O_13_ | 341.0660, 301.0339, 300.0275, 271.0248, 257.0454, 151.0026, 107.0128 | ^3#^Carboxymethyl quercetin-O-hex |
|  | 6.70 | 193.0498 | C_10_H_9_O_4_ | 175.0235, 131.0336 | ^2^Ferulic acid |
|  | 6.73 | 275.0559 | C_14_H_11_O_6_ | 247.0247, 231.0656, 163.0754, 125.0229 | ^2^Teadenol A/B |
|  | 6.76 | 417.0827 | C_20_H_17_O_10_ | 357.0610, 327.0511, 285.0381, 233.0450, 139.0389, 125.0231 | ^3#^Kaempferol-C-pen |
|  | 6.76 | 303.0511 | C_15_H_11_O_7_ | 285.0404, 149.0231, 151.0026, 125.0230 | ^1^Taxifolin |
|  | 6.80 | 901.2413 | C_42_H_45_O_22_ | 609.1453, 447.0922, 301.0350, 271.0253, 151.0024 | ^2^Quercetin-O-Co-hex-di-dhex |
|  | 6.81 | 593.0930 | C_29_H_21_O_14_ | 423.0733, 331.0446, 289.0721, 271.0615, 245.0819, 169.0132, 125.0231 | ^2^ (-)-Epicatechin 3, 5-di-O-gallate |
|  | 6.84 | 583.1095 | C_28_H_23_O_14_ | 463.0876, 371.0757, 341.0670, 301.0346, 169.0133, 137.0226, 125.0231 | ^3#^Quercetin-O-hex-4-hydroxybenzoic acid |
|  | 6.85 | 425.0881 | C_22_H_17_O_9_ | 355.0802, 273.0771, 255.0661, 229.0863, 169.0132, 125.0231 | ^2^Epiafzelechin-gallate |
|  | 6.88 | 887.2259 | C_41_H_43_O_22_ | 432.1011, 301.0349, 285.0404, 271.0254, 163.0391, 151.0020, 137.0228 | ^2^Quercetin-O-Co-pen-dhex-hex |
|  | 6.91 | 885.2466 | C_42_H_45_O_21_ | 431.0971, 285.0407, 255.0317, 163.0386, 145.0283, 125.0232 | ^3#^Kaempferol-O-Co-hex-di-dhex |
|  | 6.94 | 399.0721 | C_20_H_15_O_9_ | 381.0584, 233.0450, 215.0342, 189.0185, 125.0230 | ^2^Theaﬂagallin |
|  | 6.96 | 435.1086 | C_24_H_19_O_8_ | 341.0663, 289.0721, 217.0135, 177.0182, 109.0279 | ^2^Epicatechin-[8, 7-e]-4β-(4-Hydroxyphenyl) 3, 4-2H-2 (3H)- pyrone-1 |
|  | 7.0 | 431.0983 | C_21_H_19_O_10_ | 341.0661, 311.0559, 285.0400, 191.0336, 125.0227 | ^2^Kaempferol-C-dhex |
|  | 7.05 | 595.1672 | C_27_H_31_O_15_ | 415.1034, 385.0924, 355.0817, 313.0713, 271.0612, 125.0232 | ^3#^Naringenin-C-di-hex |
|  | 7.09 | 489.1042 | C_23_H_21_O_12_ | 191.0552, 179.0342, 163.0391, 151.0392 | ^2^Gallic acid-p-CoQA |
|  | 7.10 | 871.2312 | C_41_H_43_O_21_ | 341.0660, 313.0697, 285.0405, 257.0465, 163.0387, 145.0282, 125.0229 | ^3#^Kaempferol-O-Co-pen-dhex-hex |
|  | 7.11 | 755.1829 | C_36_H_35_O_18_ | 301.0350, 271.0251, 255.0296, 163.0391, 151.0025, 145.0283 | ^2^Quercetin-O-Co-dhex-hex |
|  | 7.17 | 609.1253 | C_30_H_25_O_14_ | 301.0349, 289.0719, 271.0256, 178.9977, 151.0024, 125.0230 | ^2^Theasinensin C |
|  | 7.17 | 317.0302 | C_15_H_9_O_8_ | 289.0366, 271.0246, 227.0343, 178.9976, 151.0025, 137.0231, 107.0124 | ^1^Myricetin |
|  | 7.18 | 499.1250 | C_25_H_23_O_11_ | 191.0552, 179.0341, 163.0389, 125.0229 | ^2^1-C-3-p-CoQA |
|  | 7.18 | 451.1036 | C_24_H_19_O_9_ | 341.0663, 311.0569, 289.0708, 217.0135, 179.0335, 161.0233, 109.0282 | ^2^ (Epi)catechin 3-O-caﬀeoate |
|  | 7.27 | 447.0935 | C_21_H_19_O_11_ | 301.0341, 300.0274, 161.0446, 101.0230 | ^2^Quercetin-3-O-α-L-rhamnoside |
|  | 7.23 | 331.0819 | C_17_H_15_O_7_ | 289.0719, 245.0817, 205.0498, 125.0231 | ^3#^Acetylated (epi)catechin |
|  | 7.24 | 901.2413 | C_42_H_45_O_22_ | 609.1453, 447.0922, 301.0350, 271.0253, 151.0024 | ^2^Quercetin-O-Co-hex-di-dhex |
|  | 7.34 | 401.0878 | C_20_H_17_O_9_ | 341.0661, 311.0559, 269.0455, 233.0449 | ^3#^Apigenin-C-pen |
|  | 7.38 | 739.1889 | C_36_H_35_O_17_ | 285.0404, 257.0456, 229.0499, 163.0386, 145.0284, 125.0229 | ^3#^Kaempferol-O-Co-dhex-hex |
|  | 7.38 | 885.2466 | C_42_H_45_O_21_ | 431.0980, 285.0407, 255.0317, 163.0386, 145.0283, 125.0232 | ^3#^Kaempferol-O-Co-hex-di-dhex |
|  | 7.41 | 609.1253 | C_30_H_25_O_14_ | 301.0349, 289.0719, 271.0256, 178.9977, 151.0024, 125.0230 | ^2^Theasinensin C isomer |
|  | 7.43 | 287.0557 | C_15_H_11_O_6_ | 245.0812, 151.0025, 135.0439, 107.0125 | ^2^Eriodictyol |
|  | 7.43 | 725.1722 | C_35_H_33_O_17_ | 285.0405, 257.0454, 229.0499, 169.0130, 125.0232 | ^3#^Rutin derivative |
|  | 7.57 | 593.1304 | C_30_H_25_O_13_ | 447.0931, 285.0403, 257.0458, 229.0502, 163.0390, 151.0024 | ^3#^Kaempferol-O-Co-hex |
|  | 7.60 | 435.1086 | C_24_H_19_O_8_ | 341.0663, 289.0721, 217.0135, 177.0182, 109.0279 | ^2^Epicatechin-[8, 7-e]-4β-(4-Hydroxyphenyl) 3, 4-2H-2 (3H)- pyrone-2 |
|  | 7.62 | 755.1829 | C_36_H_35_O_18_ | 301.0350, 271.0251, 255.0296, 163.0391, 151.0025, 145.0283 | ^2^Quercetin-O-Co-dhex-hex |
|  | 7.69 | 431.0983 | C_21_H_19_O_10_ | 341.0661, 311.0559, 285.0400, 191.0336, 125.0227 | ^2^Kaempferol-C-dhex |
|  | 7.70 | 483.1297 | C_25_H_23_O_10_ | 337.0937, 191.0542, 173.0445, 163.0390, 119.0488 | ^2^1, 3/3, 5-di-p-CoQA |
|  | 7.73 | 481.1143 | C_25_H_21_O_10_ | 289.0730, 233.0450, 193.0136, 169.0131, 125.0230 | ^2^ (-)-Epigallocatechin 3-O-ferulate |
|  | 7.80 | 593.1304 | C_30_H_25_O_13_ | 447.0931, 285.0403, 257.0458, 229.0502, 163.0390, 151.0024 | ^3#^Kaempferol-O-Co-hex |
|  | 7.81 | 481.1143 | C_25_H_21_O_10_ | 289.0730, 233.0450, 193.0136, 169.0131, 125.0230 | ^2^ (-)-Epigallocatechin 3-O-ferulate isomer |
|  | 7.89 | 201.1125 | C_10_H_17_O_4_ | 183.1018, 139.1116, 111.0803 | ^2^Sebacic acid |
|  | 7.94 | 137.0233 | C_7_H_5_O_3_ | 119.0124, 109.0280, 93.0331 | ^2^Salicylic acid isomer |
|  | 8.02 | 285.0403 | C_15_H_9_O_6_ | 257.0453, 241.0505, 229.0503, 151.0026, 107.0124 | ^1^Luteolin |
|  | 8.04 | 287.0557 | C_15_H_11_O_6_ | 245.0812, 151.0025, 135.0439, 107.0125 | ^2^Eriodictyol isomer |
|  | 8.09 | 331.0458 | C_16_H_11_O_8_ | 315.0512, 300.0272, 271.0252, 179.0333, 181.0134, 151.0025 | ^3#^3, 5, 7, 3’, 4’-Penhydroxy-5’-methoxyflavone isomer |
|  | 8.12 | 301.0354 | C_15_H_9_O_7_ | 273.0405, 193.0141, 151.0025, 121.0281, 107.0124 | ^1^Quercetin |
|  | 8.12 | 380.9919 | C_15_H_9_O_10_S | 301.0352, 273.0405, 257.0456, 193.0141, 151.0025, 121.0281 | ^2^Quercetin sulfide |
|  | 8.41 | 396.9869 | C_15_H_9_O_11_S | 317.0300, 289.0366, 271.0246, 227.0343, 178.9976, 151.0025, 137.0231, 107.0124 | ^2^Myricetin sulfide |
|  | 8.71 | 593.1304 | C_30_H_25_O_13_ | 447.0931, 285.0403, 257.0458, 229.0502, 163.0390, 151.0024 | ^3#^Kaempferol-O-Co-hex |
|  | 8.87 | 215.1282 | C_11_H_19_O_4_ | 197.1174, 153.1273, 73.0755 | ^2^Undecanedioic acid |
|  | 9.04 | 269.0454 | C_15_H_9_O_5_ | 225.0546, 201.0540, 151.0025, 107.0123 | ^1^Apigenin |
|  | 9.13 | 271.0609 | C_15_H_11_O_5_ | 227.0711, 177.0183, 151.0025, 119.0489, 107.0124, 93.0331 | ^2^Naringenin |
|  | 9.27 | 299.0559 | C_16_H_11_O_6_ | 284.0329, 271.0251, 255.1023, 151.0026, 121.0281 | ^2^3', 4', 5-Trihydroxy-7-methoxyflavone |
|  | 9.32 | 285.0403 | C_15_H_9_O_6_ | 257.0460, 229.0501, 169.0647, 151.0027 | ^1^Kaempferol |
|  | 9.48 | 315.0509 | C_16_H_11_O_7_ | 300.0274, 271.0237, 255.0271, 151.0025, 107.0129 | ^3#^Isorhamnetin |
|  | 9.49 | 739.1670 | C_39_H_31_O_15_ | 285.0405, 255.0299, 187.0387, 163.0387, 145.0282, 119.0487 | ^3#^Kaempferol-O-hex-di-Co |
|  | 9.71 | 299.0559 | C_16_H_11_O_6_ | 284.0329, 271.0251, 255.1023, 151.0026, 121.0281 | ^2^3', 4', 7-Trihydroxy-5-methoxyflavone |

C, caﬀeoyl; p-Co, p-coumaroyl; Co, coumaric acid; F, feruyl; QA, quinic acid; G, galloyl; B, benzenepropanoyl; GA, gallic acid pen, pentose; dhex, deoxyhexose; hex, hexose; GDL, D-(+)-Glucono-1, 5-lactone; glc ua, Glucuronic acid;

^1^ Identiﬁed by Standard substances based on level 1.

^2^ Identified based on level 2.

^3#^ Potential new compounds identified in pu-erh tea based on level 3.

* [M+H]^+^ ion.
